# Interindividual and intraindividual differences in recovery of German junior female ice hockey players during 2020 World Championship Division IA tournament

**DOI:** 10.1038/s41598-025-09906-y

**Published:** 2025-07-15

**Authors:** Asja Kiel, Reinhold Kliegl, Annika Hof zum Berge, Karl Schwarzenbrunner, Christian Puta, Lisa Kullik, Sarah Jakowski, Michael Kellmann

**Affiliations:** 1 Faculty of Sport Science, Department of Sport Psychology, Ruhr University Bochum, Bochum, Germany; 2 Department of Sport and Health Sciences, Faculty of Human Sciences, University of Potsdam, Potsdam, Germany; 3 German Ice Hockey Federation, Munich, Germany; 4 Department of Sports Medicine and Health Promotion, Friedrich-Schiller-University Jena, Jena, Germany; 5 Center for Sepsis Control and Care, Center for Sepsis Control and Care (CSCC), Jena University Hospital/Friedrich-Schiller-University Jena, Jena, Germany; 6 Center for Interdisciplinary Prevention of Diseases related to Professional Activities, Jena, Germany; 7 School of Human Movement and Nutrition Sciences, The University of Queensland, Brisbane, Australia

**Keywords:** Psychology, Signs and symptoms

## Abstract

World Championship tournaments pose multiple possible recovery-challenging conditions in ice hockey. This investigation aimed at assessing subjective recovery patterns of the German women’s junior national ice hockey team during preparation camp and tournament of the 2020 World Championship Division IA. Players completed the German paper-pencil version of the Short Recovery and Stress Scale regularly over the 14-day survey period. The assessment analyzed potential recuperative effects of overnight recovery and recovery during the day reflected in recovery-stress states. Specifically, linear mixed modelling (LMM) was used to determine inter- and intraindividual response- and effect-profiles on recovery processes and changes over time. Three sets of contrasts were specified *à priori* for planned comparisons. Regarding main effects, results showed favorable recovery-stress states in the morning. Recovery generally seemed to decrease throughout the day and a generally higher recovery level during the tournament phase in comparison to the preparatory camp period was indicated. Results emphasized the need for considering interindividual differences concerning the perception of different recovery facets. This study could serve as a starting point for introducing the LMM approach into sports science for the examination and presentation of individual recovery and training response patterns and individual cases with recourse to group statistics.

**Supplementary Information:**

The online version contains supplementary material available at 10.1038/s41598-025-09906-y.

## Introduction

Athletes face a variety of sport-specific stressors such as competition, training, or travel as well as personal stressors such as the work-life interface^1–3^. To ensure and maintain athletes’ performance capability, well-being, and health, multifaceted stress-inducing factors need to be adequately balanced with recovery. A persistent recovery-stress imbalance could result in undesired consequences such as underrecovery and nonfunctional overreaching as common preliminary stages of the overtraining syndrome. Recovery is seen and defined as an umbrella term that can be specified by different recovery modalities such as regeneration or strategies for psychological recovery. It is further considered to be a time-bound process of restoration of multiple processes (e.g., psychological, physiological) and factors^4,5^. A series of important psychological and physiological functions which are assumed to be essential for recovery are ensured through sleep^3^.

In general, sleep is regarded as fundamental for health, sports, and exercise performance including reaction time, and attentive, learning and decision-making processes^6,7^. Potential consequences resulting from disturbed sleep such as diminished mental well-being, impaired immunological, cognitive, learning and memory consolidating processes clearly point to the need for adequate sleep and (overnight) recovery in athletes^3^. The systematic application of sleep and rest as recommended countermeasures for underrecovery and nonfunctional overreaching further illustrates the close interaction of sleep and recovery^5^. This is also present in research findings on the effects of sleep on athletes’ recovery and subsequent performance measures^8,9^. A recent study on sleep in this sample of German female youth national ice hockey athletes before and after a preparation camp preceding the 2020 World Championship Division IA tournament suggested that effects of performance demands might be reflected in recovery patterns rather than directly affecting sleep, which requires further investigation^10^. Stress, recovery, sleep, and performance demands are assumed and shown to be interrelated, but the direction, time courses, and concrete interrelations need to be further analyzed^10–12^.

There is a high interindividual variability in recovery processes and needs, sleep patterns, and sleeping behavior that should be considered when analyzing recovery and sleep in athletes^5,13^. The need for an approach that considers the individuality in athletes’ processes is also shown by inter- and intraindividual differences found for interactions between training load and the recovery-stress state, as well as interindividual differing training responses and individual adaptions that deserve particular consideration^14,15^. Furthermore, group means do not provide information on individual recovery and sleep needs, processes, and reactions to (tournament and preparation camp) conditions and circumstances^16^. The consideration of individual recovery and sleep patterns and processes is therefore highly relevant and seems to be a promising approach in recovery research.

Physical contact, speed, high physical and skill demands as well as a need for quick recovery are often named as typical characteristics of ice hockey^17,18^. This team sport is further referred to as predisposing athletes to accelerated and potentially chronic fatigue as well as to injury^17,19^. The sport is characterized by demanding conditions, a high intensity as well as tightly timed training and tournament schedules. Thus, players must quickly recover from highly intensive shifts during the game and there is a need for quick and effective post-game recovery^19,20^. The need for adequate recovery and the high risk of injury and premature fatigue indicate and underline the need for monitoring and analyzing recovery and stress states regularly in (youth) ice hockey players to prevent undetected and chronic inadequate recovery which could contribute to injury or underrecovery. According to the present state of research stated in a recent review on the physiology of ice hockey, there is a lack of sufficient evidence and a need for research on post-game recovery patterns in ice hockey as well as on recovery during periods of congested game schedules^19^.

Games and competition situations can affect sleep and recovery in multiple ways. During phases of consecutive games, impairments in athletes’ sleep quantity and quality are likely due to constant anticipation and presence of competition^1^. Comparing game and non-game days, the amount of sleep obtained after games was partially shown to be lower than non-game days’ sleep duration^21,22^. Factors such as continued arousal, post-game routines, nutrition, or elevation of physiological variables following games played late in the evening provide possible causes for altered sleeping behavior and disturbed or restricted sleep and recovery after late-night competition^1,23^. Another important factor for sleep and recovery impairments is electronic media exposure in the evening^24^. Besides the time of day as a potential sleep- and recovery-influencing factor, the location of matches was also shown to potentially cause recovery and sleep patterns’ disruption^25^. During congested competition schedules, athletes may also suffer from problems with falling asleep or other sleep disturbances since stressors and disruptions like stress-, anxiety-, and tension-related arousal are likely to be elevated prior to competition^23,26,27^.

World Championship tournaments pose a series of possible recovery- and sleep-challenging conditions with a congested schedule, a series of important games, evening and late-night games, and an immediately preceding preparation camp. Differences in recovery patterns over the course of time might result from consecutive games, associated demands, evening games, accumulation of muscle damage, fatigue (i.e., physical and mental), or stress^1,19^. Therefore, this investigation aimed to assess subjective recovery patterns of the German women’s junior national ice hockey team during preparation training camp and tournament of the 2020 World Championship Division IA. A tabular representation of the match schedule including game times and particular events can be found in *Supplementary Table **S1*. The assessment addressed the need for research on recovery patterns in ice hockey and analyzed potential recuperative effects of sleep, overnight recovery, and of recovery during the day reflected in recovery-stress states. Specifically, linear mixed modelling was used to determine inter- and intraindividual response- and effect-profiles on recovery processes and changes over time. This methodology is increasingly used in general^28–30^ and specifically in sports science^31–34^. However, by applying this methodical approach, we aimed to address inter- and intraindividual differences in recovery patterns of response- and effect-profiles in competitive sports^35^, with simultaneous consideration of courses at a group level. This was enabled using group statistics for improving the prediction for the individual athlete (borrowing strength) as a special linear mixed modelling feature.

## Results

Statistics for fixed effects of time-of-day contrasts, phase, the quadratic trends within the preparatory training camp (TC) and within the 2020 IIHF U18 World Championship Division IA tournament (WCDT), the effect of games and late games on the previous day, contrasts for the Short Recovery and Stress Scale (SRSS) items as well as the interactions are displayed in Table 1a and 1b. Estimated variance components (VCs) and associated correlation parameters (CPs) are listed in Tables 2 and 3 (see below). Reference will be made to these tables when presenting the results in the following sections. Contrast specifications and Linear Mixed Model (LMM) analyses can be found in the methods sections (Sect. “Contrast specifications” and “Linear mixed model selection”).

**Table 1:** LMM fixed-effect estimates.

Source of variance	Estimate	z-value	*p*-value
*Main effects*			
Grand Mean	3.76	28.17	**< 0.001**
Morning – night (tod2_1)	0.74	5.97	**< 0.001**
Morning – day (tod2_3)	0.37	3.30	**0.001**
Phase	0.14	2.66	**0.008**
TC (linear; tc1)	0.05	2.03	0.043
TC (quadratic; tc2)	0.05	3.64	**< 0.001**
WCDT (linear; wcdt1)	−0.04	−1.78	0.075
WCDT (quadratic.; wcdt2)	0.01	1.38	0.167
SSS – SRS (c1)	0.90	5.54	**< 0.001**
Specific – overall (c2)	0.95	9.95	**< 0.001**
Emotional – mental/physical (c3)	0.78	4.82	**< 0.001**
Mental – physical (c4)	0.01	0.07	0.944
Game day (game)	−0.06	−0.74	0.462
Day after late game (dalg)	−0.04	−0.46	0.648
***Interactions***			
tod2_1 x phase	0.27	2.70	**0.007**
tod2_1 x tc1	0.01	0.38	0.703
tod2_1 x tc2	−0.02	−1.20	0.231
tod2_1 x wcdt1	0.05	1.42	0.155
tod2_1 x wcdt2	−0.06	−3.71	**< 0.001**
tod2_1 x c1	0.68	3.62	**< 0.001**
tod2_1 x c2	−0.38	−3.29	**0.001**
tod2_1 x c3	−0.37	−3.43	**0.001**
tod2_1 x c4	0.42	3.17	**0.002**
tod2_1 x game	−0.16	−0.94	0.347
tod2_1 x dalg	−0.63	−3.51	**< 0.001**
tod2_3 x phase	0.48	4.76	**< 0.001**
tod2_3 x tc1	0.07	2.54	**0.011**
tod2_3 x tc2	0.06	3.72	**< 0.001**
tod2_3 x wcdt1	−0.02	−0.71	0.480
tod2_3 x wcdt2	−0.04	−2.90	**0.004**
tod2_3 x c1	0.09	0.53	0.596
tod2_3 x c2	−0.65	−6.17	**< 0.001**
tod2_3 x c3	0.36	3.61	**< 0.001**
tod2_3 x c4	−0.45	−3.66	**< 0.001**
tod2_3 x game	−0.59	−3.34	**0.001**
tod2_3 x dalg	−0.86	−4.61	**< 0.001**
tc1 x c1	−0.08	−1.49	0.137
tc1 x c2	−0.10	−2.99	**0.003**
tc1 x c3	−0.21	−6.49	**< 0.001**
tc1 x c4	−0.05	−1.20	0.229

*Note/abbreviations list* Table 1b. c1 = contrast 1 (SSS versus SRS). c2 = contrast 2 [specific recovery and stress dimensions (physical, mental, and emotional) versus overall dimensions]. c3 = contrast 3 (emotional versus physical and mental dimensions). c4 = contrast 4 (mental versus physical dimension ratings). dalg = day after late game. game = game day. SRS = Short Recovery Scale. SSS = Short Stress Scale. TC = preparatory training camp. tc1 *=* linear trend within training camp phase. tc2 = quadratic trend within training camp phase. tod = time of day. tod2_1 = contrast testing morning-versus-night ratings. tod3_2 = contrast testing morning-versus-day ratings. WCDT = 2020 IIHF U18 World Championship Division IA tournament. wcdt1 = linear trend within tournament phase. wcdt2 = quadratic trend within tournament phase.

**Table 2:** LMM variance components (VCs).

	Variance components
Grand mean	0.57
SSS – SRS (c1)	0.65
Specific – overall (c2)	0.38
Emotional – mental/physical (c3)	0.70
Mental – physical (c4)	0.46
Phase	0.15
TC (linear; tc1)	0.10
TC (quadratic; tc2)	0.05
WCDT (linear; wcdt1)	0.07
WCDT (quadratic; wcdt2)	0.03
Morning – night (tod2_1)	0.32
Morning – day (tod2_3)	0.22
Game day (game)	0.21
Day after late game (dalg)	0.23
tod2_1: game	0.37
tod2_3: game	0.42
tod2_1: dalg	0.32
tod2_3: dalg	0.43
Residual	1.06

*Note/abbreviations list* Table 2. c1 = contrast 1 (SSS versus SRS). c2 = contrast 2 [specific recovery and stress dimensions (physical, mental, and emotional) versus overall dimensions]. c3 = contrast 3 (emotional versus physical and mental dimensions). c4 = contrast 4 (mental versus physical dimension ratings). dalg = day after late game. game = game day. SRS = Short Recovery Scale. SSS = Short Stress Scale. TC = preparatory training camp. tc1 *=* linear trend within training camp phase. tc2 = quadratic trend within training camp phase. tod = time of day. tod2_1 = contrast testing morning-versus-night ratings. tod3_2 = contrast testing morning-versus-day ratings. WCDT = 2020 IIHF U18 World Championship Division IA tournament. wcdt1 = linear trend within tournament phase. wcdt2 = quadratic trend within tournament phase.

**Table 3:** Associated correlation parameters (CPs).

	Correlation parameters			
SSS – SRS (c1)	0.19			
Specific – overall (c2)	0.11	0.15		
Emotional – mental/physical (c3)	−0.11	0.19	0.46	
Mental – physical (c4)	−0.45	0.42	0.48	0.70

*Note/abbreviations list* Table 3. c1 = contrast 1 (SSS versus SRS). c2 = contrast 2 [specific recovery and stress dimensions (physical, mental, and emotional) versus overall dimensions]. c3 = contrast 3 (emotional versus physical and mental dimensions). c4 = contrast 4 (mental versus physical dimension ratings). SRS = Short Recovery Scale. SSS = Short Stress Scale.

### SRSS trends across days moderated by time of day

**Fig. 1:**
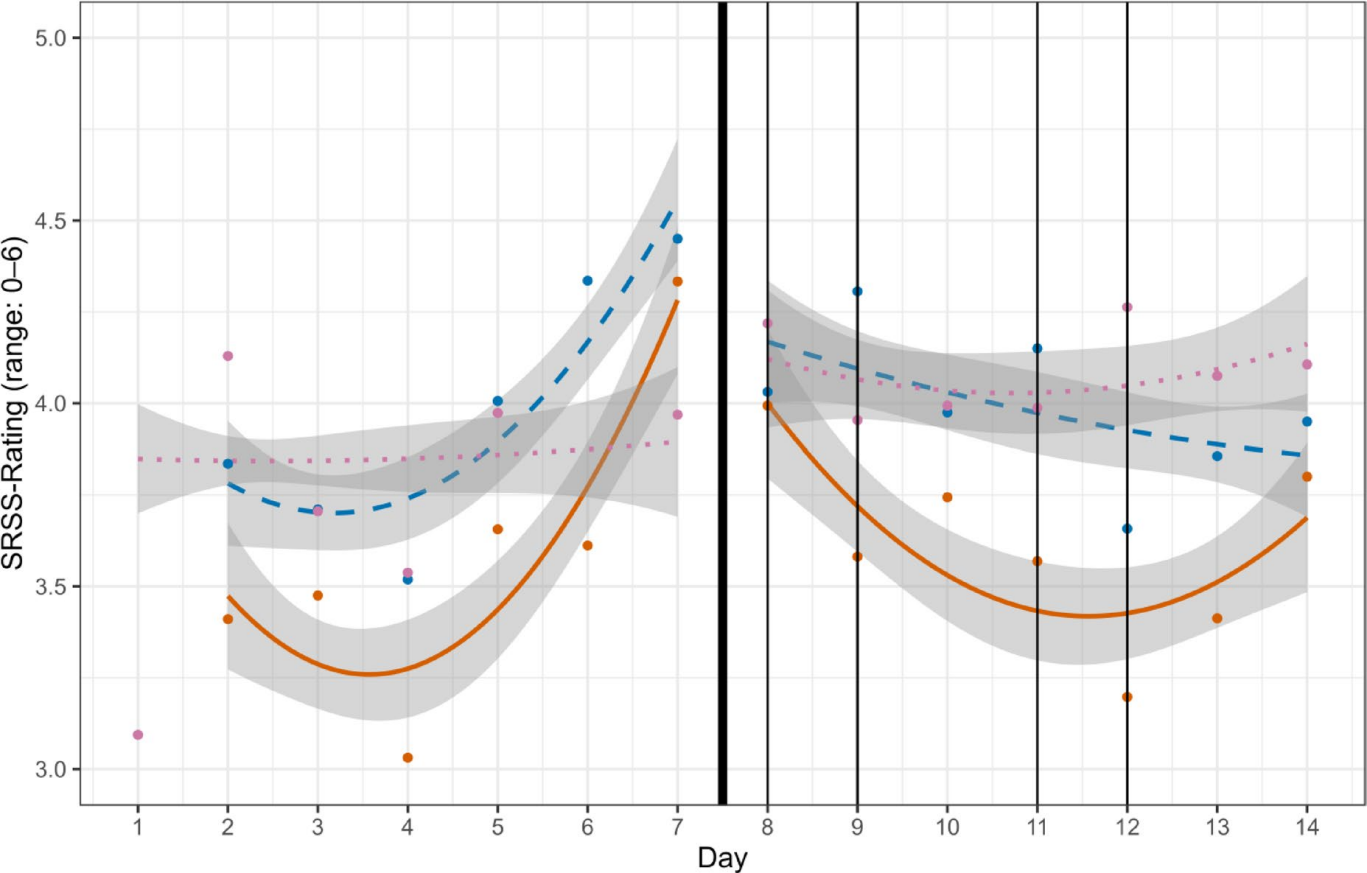
Quadratic trends across days for three time-of-day assessments for TC and WCDT phases. *Note* Fig. 1 Days 1–7: TC = preparatory training camp. Days 8–14: WCDT = 2020 IIHF U18 World Championship Division IA tournament. The thick black line separates the two phases TC and WCDT. Night scores are shifted one day forward. SRSS-Rating = Average of the eight SRSS items, dependent variable. Vertical black lines mark game days. Evening scores are marked as the orange, continuous line. Morning scores are marked as the blue, dashed line. Scores during the day are marked in pink dotted line.

Figure 1 depicts quadratic zero-order relations for the average rating of the eight SRSS items in the two phases broken down by the three daily assessments. In general, the three scores increased during the TC phase and peaked on day seven. Looking at the differences in this phase, first, as expected, SRSS scores were always higher in the morning (blue, dashed line) than the night before (orange, continuous line) and increased with a positive quadratic trend across the seven days; the interaction was not significant. The interaction was significant for the day-morning contrast due to the larger SRSS increase for morning than during-day assessments.

In the WCDT phase, SRSS ratings decreased more strongly for night than morning assessments, yielding an increase of overnight recovery (*b* = −0.06, *z =* −3.71, for the interaction of quadratic WCDT trend with first time-of-day contrast). In contrast, during-day assessment increased slightly during the WCDT phase with rating exceeding morning ratings from the 11th day on (*b* = −0.04, *z = − 2*.90, for the interaction of quadratic trend across days with second time-of-day contrast).

### (Evening) game effects

As shown in Table 1a and 1b, there were further significant fixed effects associated with effects due to having had a game in the evening of the previous day and of having played a game on the current day. For a late game on the previous day both interactions with time-of-day contrasts were significant. When there was such a game, overnight recovery was smaller (+ 0.24) compared to other days (+ 0.43); *b* = −0.63, *z* = −3.51. Recovery from morning to assessment later during the day increased on these days (+ 0.26) whereas it decreased on other days (−0.14); *b* = −0.86, *z* = −4.61.

There was also a significant interaction for game days with the morning-day time-of-day contrast (*b* = −0.59, *z* = −3.34), but this contrast was due to a spurious crossover interaction. There was no loss of goodness of fit when we took out only the ’game’-related fixed effects but kept the ’game’-related VCs in the model. The contrasts are kept in the LMM to have a base for reliable player-related individual differences of game effects.

### SRSS contrasts

#### Short Stress Scale versus Short Recovery Scale (contrast 1)

Overall ratings were more positive for (inverted) stress dimensions than recovery dimensions (*b* = 0.90, *z* = 5.54). SRSS ratings were lowest at the night before, highest in the morning, and intermediate during the day. Even though an overnight increase of the level of recovery was recognizable for both scales, the overnight increase was greater for Short Stress Scale (SSS; +0.45) than for Short Recovery Scale (SRS; +0.31); *b* = 0.68, *z* = 3.62, regarding the interaction.

#### Specific versus overall SRSS ratings (contrast 2)

**Fig. 2:**
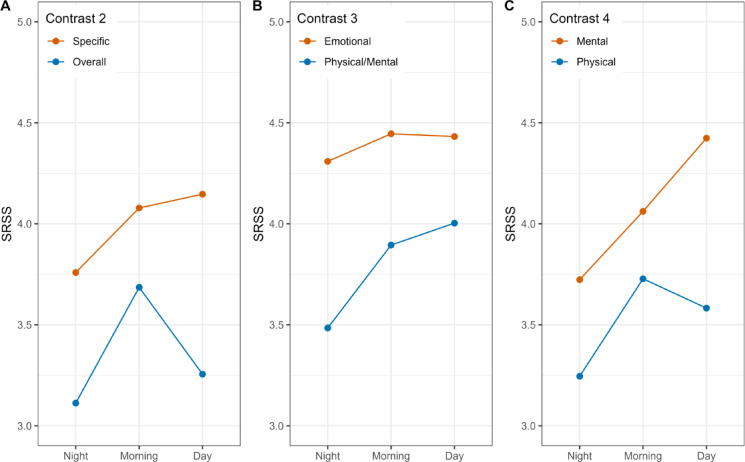
Time-of-day interactions with SSRS contrasts. *Note* Fig. 2 SRSS = Average of the eight SRSS items, dependent variable.

As shown in Fig. 2A, the level of the specific dimensions (orange line) clearly exceeded the rating for the overall dimension (blue line) (*b* = 0.95, *z* = 9.95). Overnight recovery occurred for both dimensions but was stronger for the overall rating (interaction for tod2_1: *b* = −0.38, *z* = −3.29). While there was a strong decrease from morning to day ratings for the overall dimension, the ratings for the specific dimensions even increased numerically from morning to day ratings (interaction for tod2_3: *b* = −0.65, *z* = −6.17). There was more linear change across TC days for the overall rating (interaction for tc1: *b* = −0.10, *z* = −2.99). The difference between overall and specific ratings was almost gone by the end of the TC week. The overall rating was very low on the first day.

#### Emotional versus physical/mental ratings (contrast 3)

Figure 2B displays the time-of-day interactions with this contrast. The SRSS rating for the emotional dimension (orange line) was generally higher than the physical/mental rating (blue line) (*b* = 0.78, *z* = 4.82). The contrast interacted with both time-of-day contrasts. Overnight recovery was larger for the physical/mental dimension than for the emotional (*b* = −0.37, *z* = −3.43) and there was further recovery during the day for the physical/mental dimension whereas ratings decreased for emotion (*b* = 0.36, *z* = 3.61). There was more linear change across TC days for the physical/mental dimension than for the emotional (interaction for tc1: *b* = −0.21, *z* = −6.49).

#### Mental versus physical dimension ratings (contrast 4)

Figure 2C displays the time-of-day interactions with this contrast. The overall level of recovery was numerically higher for the mental (orange line) than for the physical dimension (blue line), but the main contrast was not significant (*b* = 0.01, *z* = 0.07), presumably the high correlations with Grand Mean (GM) and other contrasts accounted for this effect (see Table 4 below). However, the interactions with time-of-day contrasts were both significant. Overnight recovery was larger for the physical recovery dimension than mental dimension rating (*b* = 0.42, *z* = 3.17) and, whereas there was some degradation of recovery from morning to day for the physical dimension rating, recovery further increased for mental dimension ratings (*b* = −0.45, *z* = −3.66).

**Table 4:** Dimensions of the Short Recovery Scale (SRS) and the corresponding dimensions of the Short Stress Scale (SSS).

SRS	SSS	Dimension
Physical Performance Capability	Muscular Stress	Physical
Mental Performance Capability	Lack of Activation	Mental
Emotional Balance	Negative Emotional State	Emotional
Overall Recovery	Overall Stress	Overall

*Note/abbreviations list* Table 4. SRS = Short Recovery Scale. SSS = Short Stress Scale.

### Variance components and correlation parameters

There are reliable player differences for 18 VCs (i.e., for fixed effects reported in Table 1; see Table 2). Table 3 displays also reliable CPs among GM and the four SRSS contrasts. Contrast c4 correlates strongly positively with the other contrasts and strongly negatively with GM; there is also a strong positive CP for c2 and c3 contrasts. Other CPs are weak and not significant when profiled (or bootstrapped). These individual differences will be used for the generation of players’ response and effect profiles.

### Response and effect profiles of individual players

In general, individual players’ patterns differed from the mean of the team for the GM and the quasi-experimental effects. Focusing on one of the most relevant quasi-experimental effects for this study’s research question, ‘tod2_1’ which illustrated recovery during sleep as the contrast between the night and morning recovery ratings, several players clearly deviated from the team’s mean value. These deviations could be explained by players’ individual profiles. There seemed to be a more pronounced difference between morning and night recovery ratings in stronger favor of recovery ratings in the morning for several players, especially for those who deviated from the team’s mean value, while for other players there were smaller or just slight differences between morning and night recovery ratings.

**Fig. 3:**
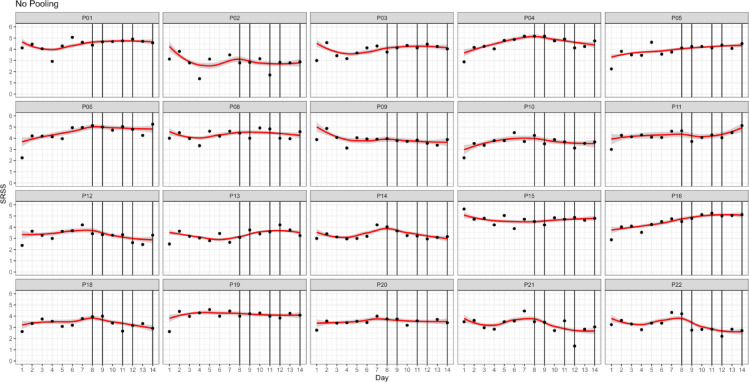
Predictions for each observation for each player with no pooling for preparatory training camp (Day 1–7) and World Championship Division IA tournament (Day 8–14). *Note* Fig. 3P = Player. Vertical lines mark game days. Black dots mark individual data of players. Red lines display predictions.

**Fig. 4:**
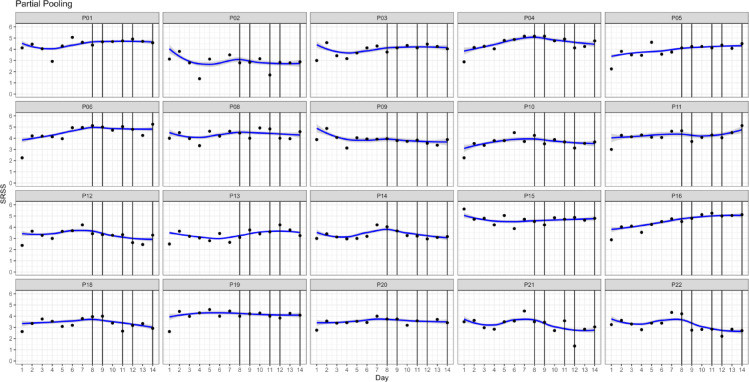
Predictions for each observation for each player with partial pooling for preparatory training camp (Day 1–7) and World Championship Division IA tournament (Day 8–14). *Note* Fig. 4P = Player. Vertical lines mark game days. Black dots mark individual data of players. Blue lines display predictions.

Figure 3 displays the generated predictions for each observation for each player using only her own data, more explicitly said not pooled across players for TC and WCDT. Partial pooling coefficients and fitted values are depicted in Fig. 4.

The second SRSS contrast (c2, specific versus overall SRSS ratings) which is presented in Fig. 2A shows a range of upward and downward deviations from the team’s mean, depending on the direction of the player’s individual deviation between the overall and the specific SRSS dimension rating. For some players, ratings on the specific dimensions’ SRSS items were higher than the rating for the overall recovery and stress dimension, other individual profiles suggested an opposed pattern.

## Discussion

The aim of this investigation was to assess subjective recovery patterns of the German women’s junior national ice hockey team during the preparation camp and tournament of the World Championship Division IA and to analyze potential effects of overnight recovery and recovery during the day reflected in perceived recovery-stress states. We used LMM to enable the determination of individual response- and effect profiles on recovery processes and changes over time. In general, results on overall changes in recovery-stress states showed favorable recovery-stress states in the morning. Regarding overnight recovery, results indicated and confirmed a recuperative effect of night sleep and its importance for recovery and regeneration^3^. Apart from the recuperative effect of night sleep and its favorable effect on the subjective level of recovery displayed in the significant main effect for the morning-night contrast, this effect might have been due to growing fatigue and an increasing need for sleep and recovery in the evening over the whole survey period. Furthermore, recovery generally seemed to decrease throughout the day which could be attributed to tournament- and training camp-associated conditions and demands. The significant main effect of the day-morning contrast might be explainable by training- and load-associated recovery reductions over the course of the day. The high intensity of training sessions and games, as well as TC and WCDT conditions, may have resulted in increased stress and fatigue throughout the day^1,19^. The significant main effect for phase indicating a generally higher recovery level during the WCDT in comparison to the TC period is potentially explainable by a successful acclimatization and preparation of players over the period of TC towards the WCDT. Furthermore, comparing the two phases, differing physical demands and stress and recovery perceptions associated with different conditions may have contributed to the difference. Furthermore, the WCDT phase might have been associated with increasing readiness to perform and the effects of game day routines which are possibly also reflected in the subjectively perceived level of recovery^5,36^.

The positive quadratic trend and increase of the subjective level of recovery over the course of the TC indicated that a preparatory TC seems to be a favorable measure before an important sport event such as the WCDT as it provides a possibility for players to acclimatize, balance stress and recovery, and prepare for the tournament mentally and physically^10^. The general trend of the increased recovery scores during the TC phase for day, morning, and night SRSS ratings depicted in the zero-order relations in Fig. 1 could potentially also be explained by the purpose of the TC of an acclimatization of players and a growing level of recovery and readiness to perform over the course of the TC towards the start of the WCDT. The interim decline of recovery levels shown in the zero-order relations’ figure might be explainable by the nomination process and associated tension, nervousness, and increased subjective effort in some players^10^. As expected, during TC, a recovery-favoring effect of sleep became apparent as depicted in Fig. 1. The development of during-day assessed SRSS ratings exceeding morning ratings from the 11th day on might indicate a growing sleep need and increasing sleep inertia towards the end of the WCDT due to accumulated fatigue and the need for quick post-game recovery^1,19^. This growing need for sleep and recovery was potentially also reflected in the stronger decrease of SRSS ratings for night than morning assessments. In contrast to expectations and courses recognizable from the figure, there was no statistical evidence for the interaction of the morning-night time-of-day contrast with the quadratic trend over the TC. The effect may not have been significant in the context of the model, as the figure depicted zero-order relations without adjustments for the other effects.

The smaller overnight increase in recovery on days following late games might have been due to shifted sleep times, potentially prolonged sleep onset, and changed sleep architecture after late night games possibly leading to sleep inertia and increased levels of sleepiness and tiredness in the morning. The increase in recovery during the day on these days could be explained by potentially diminishing sleepiness and tiredness over the course of the day due to daytime naps and disappearing sleep inertia^23,37^. These effects might have been moderated by increased self-awareness due to game-day and post-game day associated routines^38^.

The greater overnight increase in recovery level for SSS than for SRS may reflect a response bias against agreeing with negative questions. Potentially, a decrease of the amount of stress might be more noticeable than an increase in recovery dimensions as indicated by the significant main effect for the first contrast. This contrast served primarily as a control to align inverted SSS and SRS ratings.

The results for the second SRSS contrast emphasize the necessity and importance of differentiating between different dimensions of recovery [i.e., between specific recovery and stress dimensions (physical, mental, and emotional) and an overall dimension]. Discussing the results that overnight recovery was stronger for the overall dimension, as well as there was a strong decrease from morning to day ratings for the overall dimension ratings, the overall recovery dimension seemed to be more strongly affected by situational and environmental influences such as physical demands or sleep than the specific dimensions of recovery and stress. High demands and stress might rather be reflected in decreasing overall recovery levels than in specific recovery facets or dimensions. The interactions further confirm the recuperative effect of sleep through the nightly increase in recovery levels which became apparent for all recovery dimensions. The declining difference between the overall and specific dimensions by the end of the TC week might be explainable by an increasing familiarity with the SRSS as a psychometric instrument and a growing understanding of the different facets of recovery. Possibly, the repeated filling out of the questionnaire might have promoted the capability to introspection and self-awareness over the course of the survey period.

The results on the third SRSS contrast (i.e., emotional versus physical/mental ratings) indicated SRSS ratings of the emotional recovery dimension exceeded the physical/mental SRSS dimensions ratings. Potentially, TC and WCDT associated conditions (i.e., high training and game intensity, physical and mental load in training sessions and WCDT games), rather affected the physical/mental dimensions of recovery than the emotional dimension. Sleep and overnight recovery also seemed to have a stronger effect on physical and mental recovery than on the emotional dimension. This might be explainable by the assumption that due to the high training load and intensity of training sessions and games as well as due to shortened sleep length after late-night games there might have been an increased amount of deep sleep and a reduced amount of REM-sleep^26^. For the physical/mental dimension, recovery further increased throughout the day what could be attributable to decreasing sleep inertia throughout the day and increased readiness to perform of players towards evening games and afternoon training sessions. Decreasing ratings for the emotional dimension of recovery during the day might be explainable by nomination processes- and game-related arousal and tension during TC and WCDT^26^. Regarding the courses of the contrasted SRSS dimensions, the above-mentioned potential acclimatization effect of the TC might have rather been reflected in physical/mental dimensions of recovery, as there was more linear change across TC days for these dimensions than for the emotional.

The results for the fourth SRSS contrast (i.e., mental versus physical dimension ratings) confirmed the assumption that World Championship tournaments expose athletes to physical demands and stress that need to be adequately balanced with recovery^5^. Results revealed that there was greater sleep-related recovery for the physical recovery dimension. This finding could be attributed to a higher amount of deep sleep caused by high training loads resulting in greater physical recovery overnight effects to the detriment of REM sleep, which is associated with mental processes^26^. The finding of an increase for the mental recovery dimension ratings from morning to day might be explainable by dissipating sleep inertia over the course of the day^23,37^.

For some players, however, only a small difference between evening and morning ratings could be observed indicating a rather small recuperative effect of sleep and overnight recovery in these players. It must be considered that for some of these players, recovery ratings were rather high in general, so there might have been a ceiling effect. However, LMM analyses including the assessment of individual patterns and courses of recovery enabled the identification of players that did not perceive a recuperative and recovery-promoting effect overnight. These deviations and anomalies could serve as a starting point for possible adaptions of training, pre- and post-game regeneration, and personalized recovery strategies and adjustments as well as sleep management interventions. Strategies such as sleep hygiene education or relaxation techniques’ application are recommended to be implemented in these cases as well as in athletic settings to optimize individual recovery and sleep as well as sleeping behavior^39–41^.

Results for the analysis of the SRSS contrasts further emphasized the need for considering interindividual differences concerning the perception and rating of different facets of recovery. Most players seemingly perceived their mental performance capability as more pronounced than their physical performance capability. This could be explained by high physical demands induced by the high intensity of games and the congested tournament game schedule potentially having a stronger impact on the perceived physical performance capability^19^. However, some players showed a reverse pattern. These athletes might have perceived tournament conditions and games as more challenging and mentally demanding due to psychological stressors such as stress of competition, pressure to perform, handling poor performance or being judged by many sides^23,26^. Interindividual differences in players’ perceptions might also have been caused by individual load perceptions and differing position-specific demands^42,43^. As anticipated, there were differences between the TC and the WCDT phase, potentially due to different physical and mental demands. Results further provided confirmation of understanding recovery as a multidimensional and multifactorial construct and the SRSS being a suitable instrument for the assessment of different recovery facets and dimensions^5^. Future studies should consider the necessity and meaningfulness of a multidimensional assessment of recovery by appropriate instruments such as the SRSS.

Considering that the data collection took place within the scope of field research and real-life conditions, the influence of external factors’ confounding effects could not be controlled experimentally. Electronic devices use as a potential confounding and recovery-interfering factor through the inhibitory influence of blue light on melatonin secretion and arousal or alertness caused by social media activities could partly be excluded due to the non-accessibility of mobile phones during bedtime^23,44^. TV or tablet consumption during bedtime could not be excluded, thus the extent and actual existence of electronic devices’ effects on the team’s overnight recovery patterns remain open. An additional query via a sleep-behavior related questionnaire such as the athlete sleep behavior questionnaire^45^ respectively its German version^46^ could monitor if electronic devices are perceived as disturbing. Furthermore, an extension of data collection towards the end of the tournament is recommended for future research to provide further meaningful information on the potential impact of the accumulation of fatigue on recovery, post-game recovery, and recovery-stress states. The 14-day data collection period during a single tournament provides only a limited temporal window to detect stable patterns. In such a dynamic and multifactorial environment, the possibility of random fluctuations influencing the results cannot be excluded. Replication across longer timeframes or multiple competition phases would strengthen the generalizability of the findings.

Subjective data were chosen over objective measures to interfere athletes’ routines and recovery as little as possible and for being easily administrable and time effective^11,47,48^. Due to item transparency of SRSS items, manipulation of data could not completely be ruled out despite the countermeasures taken such as assuring confidential handling of all data and clarifying the survey’s purpose^49,50^. The team’s success in this tournament might have eased athlete compliance. The repeated daily assessments over 14 days represent an intensive monitoring schedule, particularly for adolescent athletes in a competitive setting. While high compliance was achieved, the potential for response fatigue or habituation effects cannot be ruled out. This is acknowledged as a methodological limitation and should be considered in future designs aiming for longer-term or high-frequency monitoring. The absence of objective sleep parameters and performance markers (e.g., physiological parameters or game-related statistics) restricts the interpretation of how individual recovery and stress states may have influenced actual performance outcomes and is therefore a key limitation of the present study. Future research should aim to integrate such objective data to more precisely assess the interplay between psychophysiological recovery processes and performance, especially in high-stakes competitive settings. Combining subjective and objective indicators would offer a more comprehensive understanding of athlete functioning and support more targeted recovery strategies. However, the subjective perception of recovery and stress is a very important component of monitoring, especially during decisive sporting events^5,51^. Nevertheless, the deduction of conclusions should be done with care considering data collection in a real-life situation and the questionnaire-based survey. The observed within-day variation in SRSS scores may partly reflect circadian fluctuations in alertness, mood, and perceived energy levels rather than solely reflecting changes in recovery status. Although the SRSS is designed to capture subjective states, we acknowledge that without controlling for time-of-day effects or collecting physiological correlates, circadian influences cannot be entirely disentangled from recovery-related changes. While an increase in SRSS scores over the course of the tournament was interpreted as a sign of improved recovery, alternative explanations are plausible. These include psychological factors such as increased competition-related motivation, growing familiarity with the questionnaire, or social desirability effects—even despite anonymity assurances. These factors could have influenced response patterns and should be considered in interpreting the results.

It should further be stressed that the results were found in this special population of German female youth national ice hockey players. The generalizability of study results to other sport types, age groups, and male athletes may be limited, since differences between male and female athletes, young and adult elite athletes, and between individual and team sport athletes may occur^52–54^.

## Methods

### Participants

Participants were 20 of the 22 female youth ice hockey athletes nominated for the final squad of the Women’s U18 German national team. Players’ age ranged from 15 to 17 with a mean age of 16.40 ± 0.68 years. Data were collected during the 14-day survey period from 2019-12-27 to 2020-01-09 which included a preparatory training camp (TC; until 2020-01-02) followed immediately by the 2020 IIHF U18 World Championship Division IA tournament (WCDT). A schematization of the study timeline, SRSS assessments and procedures can be found in *Supplementary Figure **S1*. Germany was the host of this tournament, and the TC was held at the same location (Füssen, Germany), no further (international) travel was required before tournament start. The team spent the seven-day TC in a hotel within walking distance of the ice-stadium.

On the 7th day (i.e., the day before tournament start), the team (i.e., players and the whole staff) moved to another hotel for the tournament phase. This hotel was only a few meters away from the other, so the distance and the way to the ice-stadium did not change. Conditions and sleeping accommodations were the same in both hotels. Athletes were accommodated in double rooms and a scheduled bedtime routine was implemented for all players including guided relaxation techniques (e.g., progressive muscle relaxation, dream journeys) in the evening and the collection of mobile phones during bedtime. Bedtime was normally scheduled at 22.00, except for the three evenings on which games were played at 20.00. On these evenings, bedtime was postponed to 24.00 and guided relaxation techniques were skipped as not to delay bedtime any further. Get-up times were linked to breakfast times that had to be adhered to (9.00 on late-game and off-days, 6.30 or 7.00 on days with afternoon games scheduled). Breakfast and meals were served at the hotel.

### Materials and procedure

The purpose of the TC was to provide an initial phase of acclimatization on site, to form the final tournament team, and to ensure athletes’ performance readiness for the WCDT. The tournament required optimal performance, adequate recovery-stress balances, and the ability to recover quickly from scheduled games. All athletes completed the German paper-pencil version of the Short Recovery and Stress Scale (SRSS)^55,56^ regularly over the whole survey period.

#### Short Recovery and Stress Scale

The SRSS is a sport-specific psychometric instrument developed for assessing the individual and acute recovery-stress state in athletes (see Table 4)^55,56^. The questionnaire provides a multidimensional, theoretically underpinned, economic, and change-sensitive measurement. The athlete’s current recovery-stress state on an emotional, mental, physical, and overall level is assessed by eight items. Each item is rated on a seven-point Likert scale ranging from 0 to 6 with 0 representing *does not apply at all* and 6 forming the opposite pole *fully applies*^55,56^. The Short Recovery Scale (SRS) displays the items *Physical Performance Capability* (e.g., strong), *Mental Performance Capability* (e.g., receptive), *Emotional Balance* (e.g., in a good mood), and *Overall Recovery* (e.g., rested), while Short Stress Scale (SSS) items are comprised by *Muscular Stress* (e.g., muscle soreness), *Lack of Activation* (e.g., sluggish), *Negative Emotional State* (e.g., annoyed), and *Overall Stress* (e.g., overloaded). To juxtapose physical, mental, emotional, and overall facets of recovery with the corresponding facets of stress, Table 4 illustrates the dimensions of the SRS and the appropriate dimensions of the SSS. Reported internal consistencies of α = 0.72 (SRS; for the English version α = 0.84) and α = 0.75 (SSS; for the English version α = 0.78) were satisfactory for both scales^55,56^. Construct validity was verified in studies assessing the items’ stability and intercorrelations^55–57^. The completion time of 40 to 60 s decreases with increasing familiarity with the questionnaire over time. The SRSS was chosen due to its brevity, sport-specific relevance, and ability to capture both stress and recovery dimensions with minimal burden on athletes. The instrument is well-suited for high-frequency assessments in applied sport settings, particularly during competition phases when compliance is critical.

#### Procedure

The study and investigation aimed at monitoring the recovery-stress states of the team to identify potential imbalances and problems as early as possible and allow targeted adjustments in training intensity. The purpose of the study as well as the procedure and function of monitoring processes and data collection were introduced and presented on site to all players and staff members on the arrival day of the TC. The obligation of professional confidentiality and pseudonymization of data (collection) were explained and assured to all athletes, to remove potential concerns, reduce distortion of data, and increase compliance as far as possible. The SRSS was filled in three times a day, one to two times during the day (mainly before or after training sessions) as well as every evening before going to sleep and every morning after getting up. Specifically, there were SRSS ratings from 14 days and three times of day (morning, day, evening) except for day one and day 14. On day one, which was the arrival day, players arrived around noon, so there were only SRSS ratings for the day and evening, while on day 14, which was the day of the final and deciding game against Japan, there were only ratings for the morning and day.

During TC, one to two training sessions per day were scheduled, except for two days on which test games were played which replaced respective training sessions and there was one training-free off-day. The German team played five games in the tournament^58^. *Supplementary Table **S1* displays the game plan for the German team with exact time and results^59^. They won the tournament and were promoted to the top-division^60^. All procedures were in accordance with the ethical standards of the institutional research committee and with the 1964 Helsinki Declaration and its later amendments or comparable ethical standards. Ethical approval was obtained by the ethical committee of the Faculty of Sport Science of the Ruhr University Bochum prior to the assessment (EKS V 27/2019; 2019-11-05). Written informed consent was obtained from all individual participants included in the study and parents of minor athletes.

### Statistical analysis

Pre- and post-processing of data were carried out in the R environment of statistical computing using the *tidyverse*, *haven* and *summarytools* packages^61–63^. For statistical inference, LMM analyses were conducted using the *lme4*^64^ and *remef* packages^65^.

#### Transformations

Higher SRSS ratings indicate stronger degrees of recovery for SRS items and higher stress for SSS items. To improve interpretability as well as to handle built-in negative correlations, SSS items were inverted to have all eight items and subscales correlate positively. Moreover, daily evening ratings were shifted one day forward (beyond midnight) to estimate overnight recovery in a contrast between SRSS morning rating and the last SRSS evening rating. The dependent variable was the rating of the eight SRSS items. Therefore, we neither computed an average score nor independent index scores of stress and recovery, but with the orthogonal set of contrasts we were able to take simultaneously into account the inherent SRSS multidimensionality. In our case, this procedure was mathematically necessary to harmonize the directionality of item scores for aggregated analyses. It is important to emphasize that this procedure served to demonstrate the current statistical approach.

#### Contrast specifications

Three sets of contrasts were specified *à priori* for planned comparisons^66^: (1) time-of-day (tod; three levels to two contrasts), and (2) TC and WCDT days (i.e., contrast for phase, linear quadratic trends within each of the two 7-day phases, two dummy contrasts for game day and days after an evening game during WCDT days), and (3) SSRS (eight items to four contrasts).

1. *Time-of-day contrasts.* For the three levels of time-of-day (night [i.e., “after midnight” – see above], morning, and rest of day) two contrasts were specified to test morning-versus-night ratings and morning-versus-day ratings.
2. *Day-within-phase contrasts.* In addition to the main effect of phase (i.e., TC – WCDT), changes in the TC phase (day 1–7) and in the WCDT phase (day 8–14) were each modelled with linear and quadratic trends. In addition, within the WCDT phase, 0/1-dummy codes were included for game days and for days when a game was played on the previous evening.
3. *SSRS contrasts.* Based on theoretical considerations, the contrast matrix for SRSS comprised the following four orthogonal contrasts: (c1) SSS versus SRS, (c2) specific recovery and stress dimensions (physical, mental, and emotional) versus overall dimensions, (c3) emotional versus physical and mental dimensions, (c4) mental versus physical dimension ratings. For contrasts c2, c3, and c4, ratings were averaged over corresponding items in SRS and SSS.

#### Linear mixed model selection

Statistical inferences were based on an LMM. Contrasts were included as fixed effects as described above. All of them varied within subjects, that is, in principle, they could also be included as variance components (VCs) along with their correlation parameters (CPs) in the random effect structure (RES). The full model, however, was overparameterized, preventing inferences about and interpretations of VCs and CPs. At the same time, VCs and CPs supported by the data must be included to guard against false-positive fixed effects^67^. Therefore, we followed the recommendations by Bates et al.^68^ to determine the RES of a parsimonious LMM. The initial RES was assembled from the above contrasts and associated theoretically motivated interactions. The overparameterized LMM provided an upper boundary of model complexity. As we had no theoretically motivated hypotheses for CPs, we used the conservative LRT-based BIC criterion to test whether removing terms incurred a significant loss in goodness of fit (i.e., the BIC should decrease by at least five units for the less complex LMM). Forcing all CPs except those between GM and SRSS contrasts to zero did not lead to a loss in goodness of fit (i.e., the BIC was larger, not smaller). The selection procedure was documented in the *Supplementary Script S1.*

The selected LMM comprised the following fixed effects: (1) two time-of-day contrasts, (2) quadratic polynomial contrasts for days within TC and within WCDT; two dummy contrasts for game day and evening game on the previous day for WCDT, and (3) four SRSS contrasts. Finally, the model included fixed effects for interactions of the time-of-day contrasts with all other contrasts and the interaction of SRSS contrasts with linear change in the TC phase.

A two-sided z-value of > 2.5 was used as a significance criterion for the interpretation of fixed effects. Correlation parameters were interpreted according to the classification of Cohen^69^ with *r* =.10 being classified as weak, 0.30 as medium, and 0.50 as strong correlations.

#### LMM-based ’player-level’ predictions

Parameters of the selected final model were used to compute ’player-level’ predictions for [some of] the significant effects (i.e., conditional means given the model parameters and the player’s data). The goal was to check whether there was a noticeable clustering of players with respect to their conditional means.

## Conclusions

The results demonstrate the potential of LMM as a statistical method enabling the consideration of inter- and intraindividual differences concerning the perception and rating of different facets of recovery or other psychometric and training data. The study stresses the necessity to consider individual responses to potentially recovery-affecting situations and conditions. Methodically, this is a new perspective. A particular feature of LMMs is the use of group statistics to improve predictions for the individual. The LMM approach enables the determination of individual response- and effect-profiles suitable for the development of individualized interventions (e.g., training, recovery, sleep, communication), which in turn can induce phenotypic changes. Thus, the novel approach is of particular relevance for sports science as it allows a new form of training control. This study could serve as a starting point for introducing this approach into sports science and research as a promising approach for the examination and presentation of individual sleep, recovery and training response patterns, and individual cases with recourse to group statistics.

Since there is little evidence on subjectively perceived recovery and overnight changes in recovery-stress states of female youth elite ice hockey national players, the study provides high-quality data. This survey during preparation camp and the tournament of the 2020 IIHF U18 Women’s World Championship Division IA is a unique study, since it rarely can be repeated in this form. The results underline the importance of a continuous monitoring of recovery in female youth elite ice hockey athletes as it allows an early detection of recovery-stress imbalances which in turn might prevent further accumulated negative impacts on sleep, recovery, and performance capability.

## Electronic supplementary material

Below is the link to the electronic supplementary material.

Supplementary Material 1

Supplementary Material 2

## Data Availability

The data that support the findings of this study are available on request from the corresponding author [AK]. The data are not publicly available due to their containing information that could compromise the privacy of research participants.
