# Supplementary material for: Interindividual and intraindividual differences in recovery of German junior female ice hockey players during 2020 World Championship Division IA tournament
Source: Sci Rep. 2025 Jul 15;15:25520. doi: 10.1038/s41598-025-09906-y (PMC12264116; doi:10.1038/s41598-025-09906-y)

Interindividual and intraindividual differences in recovery of German junior female ice hockey players during 2020 World Championship Division IA tournament

Supplementary Table S1

German national team during the 2020 IIHF U18 World Championship Division IA Tournament

**Date**	**Time**	**Game**	**Result**	**Remark**	**Classification**
2020-01-03	16.30	ITA-GER	0:5		
2020-01-04	20.00	GER-DEN	3:0		late-night game
2020-01-05			*game-free*		
2020-01-06	20.00	GER-HUN	2:1	overtime and shootout	late-night game
2020-01-07	20.00	GER-FRA	6:0		late-night game
2020-01-08			*game-free*		
2020-01-09	18.00	JPN-GER	1:2		

Note/abbreviations list Table S1. DEN=Denmark. FRA=France. GER=Germany. HUN=Hungary. ITA=Italy. JPN=Japan. Official IIHF game plan for the German team [59].

Supplementary Figure S1

Schematization of the study timeline, SRSS assessments and procedures

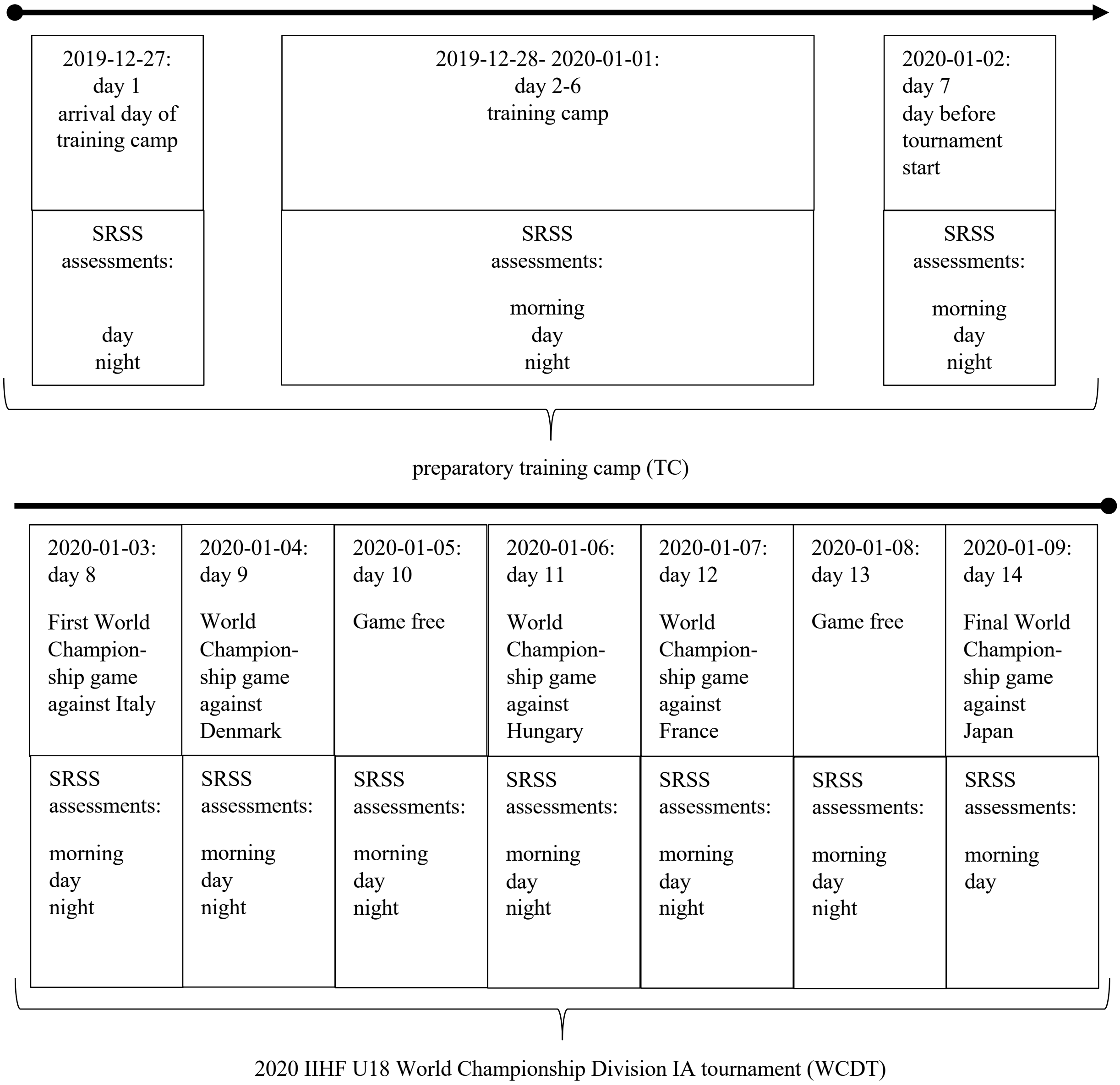

Supplement: Supplementary file 1 — Supplementary Material 1 [file 41598_2025_9906_MOESM1_ESM.pdf]
