# Supplementary material for: Interindividual and intraindividual differences in recovery of German junior female ice hockey players during 2020 World Championship Division IA tournament
Source: Sci Rep. 2025 Jul 15;15:25520. doi: 10.1038/s41598-025-09906-y (PMC12264116; doi:10.1038/s41598-025-09906-y)
Supplement: Supplementary file 2 — Supplementary Material 2 [file 41598_2025_9906_MOESM2_ESM.pdf]

# Interindividual and intraindividual differences in recovery of German junior female ice hockey players during 2020 World Championship Division IA tournament

Supplementary Script S1

*Documentation of the LMM selection procedure including the code and the results*

Asja Kiel, Reinhold Kliegl, et al.

2025-05-23

# Notes

- We refit all LMMs without cubic terms – phases are too short to yield interpretable trends
- We drop c5 to c7; they are not reliable because they contrast only two items each
- There is a built in negative correlation between SRS and SSS. Flip SSS to have all scale correlate positively. Dataframe `dat` uses original SSS ratings; dataframe `dat2` uses reflected SSS ratings.
- We flip signs of Phase and use tod2_3 (instead of tod3_2) to obtain positive main effects; simplifies interpretation of CPs, too. (We also flip c7, but this one is no longer in the LMM.)
- For the test the effect of recovery in `ToD`, we shift "Abend" one day forward (beyond midnight), both for KEB and RPE. This way, the contrast `KEB Morgen-KEBAbend` estimates recovery in the LMM. (`wcdt_day` also needs to be shifted.) this transformation causes missing values for IVs on day 8. They are all legit zeros.

## Design

During junior world championship (JWC) and during the preceding training camp, rating of sleep quality was collected once a day and ratings on the short recovery and short stress scale three times every day.

- Total sample: 22 athletes (M age = 16,36, SD= 0,73); nominated for JWC (subsample): 20 athletes (M age = 16,4, SD= 0,68)
- Dates: 2019.12.27 - 2020.01.09 (14 days, 13 nights: 7 days training camp, 7 days JWC)

- Training camp: 7 days (2019.12.27 - 2020.01.02) – incl. a rest day and two test games
- 2020 IIHF Ice Hockey U18 Women-Word Championship Div. IA
    - 7 days (2020.01.03 – 2020.01.09) – 5 WM games
    - 01.03. 16:30 Uhr ITA-GER 0:5
    - 01.04. 20:00 Uhr GER-DEN 3:0
    - 01.05. no game
    - 01.06. 20:00 Uhr GER-HUN 2:1 after overtime and shootout
    - 01.07. 20:00 Uhr GER-FRA 6:0
    - 01.08. no game
    - 01.09. 18:00 Uhr JPN-GER 1:2
- Monitoring components:
    - Baseline: EBF (77 Items), AEB, KEB, PSQI, ESS, FSVS

    - Post-TL: EBF (77 Items), KEB, PSQI, ESS, FSVS
    - KEB1 to KEB4: Short Recovery Scale (SRS)
    - KEB5 to KEB8: Short Stress Scale (SSS)
    - RPE
    - Regeneration measures

    - Evening and Morgenprotokoll + KEB (regelmäßig über den gesamten Zeitraum)

# Setup

```
library(easystats)

# Attaching packages: easystats 0.7.4 (red = needs update)
✖ bayestestR  0.15.3   ✔ correlation 0.8.7
✖ datawizard  1.0.2    ✔ effectsize  1.0.0
✖ insight     1.2.0    ✖ modelbased  0.10.0
✖ performance 0.13.0   ✖ parameters  0.25.0
✔ report      0.6.1    ✔ see         0.11.0

Restart the R-Session and update packages with `easystats::easystats_update()
`.

library(lme4)

Loading required package: Matrix

# install.packages("devtools"); devtools::install_github("hohenstein/remef")
library(remef)
```

```
Attaching package: 'remef'

The following object is masked from 'package:insight':

    has_intercept
```

```
library(tidyverse)
```

```
── Attaching core tidyverse packages ──────────────────────── tidyverse 2.0.0 ──
✔ dplyr     1.1.4     ✔ readr     2.1.5
✔ forcats   1.0.0     ✔ stringr   1.5.1
✔ ggplot2   3.5.2     ✔ tibble    3.2.1
✔ lubridate 1.9.4     ✔ tidyr     1.3.1
✔ purrr     1.0.4
── Conflicts ────────────────────────────────────────── tidyverse_conflicts() ──
✖ tidyr::expand()  masks Matrix::expand()
✖ dplyr::filter()  masks stats::filter()
✖ dplyr::lag()     masks stats::lag()
✖ tidyr::pack()    masks Matrix::pack()
✖ purrr::partial() masks remef::partial()
✖ tidyr::unpack()  masks Matrix::unpack()
ℹ Use the conflicted package (<http://conflicted.r-lib.org/>) to force all c
onflicts to become errors
```

```
library(haven)
library(cowplot)
```

```
Attaching package: 'cowplot'

The following object is masked from 'package:lubridate':

    stamp
```

```
cbPalette <- c( "#0072B2", "#D55E00", "#009E73", "#CC79A7",
                "#F0E442", "#56B4E9", "#999999", "#E69F00")

setwd("/Users/reinholdkliegl/Library/CloudStorage/Dropbox/Bochum_JWM2019")
dat2 <- readRDS("./data/JWM2019_v3.rds" ) # get preprocessed data
load(file="./fits/LMMs_JWM2019_v3.rda")
# load saved model fits, that were saved with:
#save(ovi_m2, zcp_m1, zcp_m2, prm_m1, prm_m2, prm_m3, cpx_m1,
#      file="./fits/LMMs_JWM2019_v3.rda")
```

# Contrasts

Life starts with contrasts…

For this study, treating KEB as a factor with 8 levels, we can have both crossed and nested designs. The matrix we use in the R chunk is a set of orthogonal **nested** contrasts. They are based on theoretical considerations. This is not the only option. For example, one could argue that contrasts 5, 6, and 7 represent a SRS/SSS (2) x Body/Mind (2) design with the seven contrasts. Then, the specification would be changed as shown here:

```
 -1/2, +1/2,    0,    0, -1/2, +1/2,    0,    0,  # Mind - body
 +1/2, +1/2,    0,    0, -1/2, -1/2,    0,    0,  # Subscale for Body+Mind
 -1/2, +1/2,    0,    0, +1/2, -1/2,    0,    0,  # Interaction (subscale x b
x mind)
```

This version, however, assumes that "Körperliche Leistungsfähigkeit" is equivalent to negative version of "Muskuläre Beanspruchung" and "Mentale Leistungsfähigkeit" is equivalent to a negative version of "Aktivierungsmangel". This seemed to be a bit of a stretch.

**The contrast specifications and transformations in the following chunk are already in dataframe `dat2`. The chunk is included here as reference.**

```
# This is an orthogonal set of contrasts for the eight KEB items
contr.Item <-
matrix(c(-1/4, -1/4, -1/4, -1/4, +1/4, +1/4, +1/4, +1/4,  # SSS-SRS
         +1/6, +1/6, +1/6, -1/2, +1/6, +1/6, +1/6, -1/2,  # Spec-gen (g),
         -1/4, -1/4, +1/2,    0, -1/4, -1/4, +1/2,    0,  # Emotion-(body+min
d)2
         +1/2, +1/2,    0,    0, -1/2, -1/2,    0,    0,  # SRS_bm-SSS_bm
         -1/2, +1/2,    0,    0,    0,    0,    0,    0,  # Mind-body | SRS
            0,    0,    0,    0, -1/2, +1/2,    0,    0,  # Mind-body | SSS
            0,    0,    0,   +1,    0,    0,    0,   -1), ncol=7) # SRS_g-SSS
_g
contrasts(dat2$Item) <-  contr.Item

contrasts(dat2$ToD) <- MASS::contr.sdif(3)
# tod2_1: morning-night; tod2_3: day-morning, see flip of sign below

dat2$Phase <- factor(ifelse(dat2$day <= 7, "TC", "WCDT"))
contrasts(dat2$Phase) <- (-1)*contr.sum(2)
# WCDT - TC; we flip sign to get positive estimate

mm <- model.matrix(~ 1 + Phase + Item + ToD, data=dat2 )
dat2$GM    <- mm[,  1]
dat2$phase <- mm[,  2]

dat2$c1    <- mm[,  3] # SSS - SRS
dat2$c2    <- mm[,  4] # Specific - general,
dat2$c3    <- mm[,  5] # Emotion - (body+mind)/2
```

```
dat2$c4    <- mm[,  6] # SRS_bodymind - SSS_bodymind
dat2$c5    <- mm[,  7] # Mind-body | SRS
dat2$c6    <- mm[,  8] # Mind-body | SSS
dat2$c7    <- mm[,  9] # SRS_g - SSS_ge # c1; inverted  positive effect

dat2$tod2_1  <- mm[, 10] # Time of day: morning - night
dat2$tod2_3  <- (-1)*mm[, 11]
# Time of day: morning - day # flip sign to get positive estimate

dat2$tc1 <-  ifelse(dat2$day <= 7, dat2$day -  4, 0)
# nested and centered within tc phase
dat2$tc2 <- dat2$tc1^2
dat2$tc3 <- dat2$tc1^3

dat2$wcdt1 <-  ifelse(dat2$day  > 7, dat2$day - 11, 0)
# nested and centered within wcdt phase

dat2$wcdt2 <- dat2$wcdt1^2
dat2$wcdt3 <- dat2$wcdt1^3
```

# LMM varying only intercepts

```
# sort of a minimal LMM
ovi_m1 <- lmer(keb ~ 1 + (tod2_1 + tod2_3)*(phase + tc1+wcdt1 +
                          c1+c2+c3+c4 + game+dalg) +
                         (c1+c2+c3+c4)*tc1 + (1 | Player),
                dat=dat2, REML=FALSE, control=lmerControl(calc.derivs = FALSE
))

# add quadratic trends
ovi_m2 <- lmer(keb ~ 1 + (tod2_1 + tod2_3)*(phase +  tc1+tc2 + wcdt1+wcdt2 +
                          c1+c2+c3+c4 + game+dalg) +
                         (c1+c2+c3+c4)*tc1 + (1 | Player),
                dat=dat2, REML=FALSE, control=lmerControl(calc.derivs = FALSE
))


# add quadratic trends for tc2*Item
ovi_m3 <- lmer(keb ~ 1 + (tod2_1 + tod2_3)*(phase+ tc1+tc2 + wcdt1+wcdt2 +
                          c1+c2+c3+c4 + game+dalg) +
                         (c1+c2+c3+c4)*(tc1+tc2) + (1 | Player),
                dat=dat2, REML=FALSE, control=lmerControl(calc.derivs = FALSE
))

# add quadratic trends for wcdt*Item
ovi_m4 <- lmer(keb ~ 1 + (tod2_1 + tod2_3)*(phase+ tc1+tc2 + wcdt1+wcdt2 +
                          c1+c2+c3+c4 + game+dalg) +
                         (c1+c2+c3+c4)*(tc1+tc2+wcdt1+wcdt2) + (1 | Player),
                dat=dat2, REML=FALSE, control=lmerControl(calc.derivs = FALSE
```

```
))

anova(ovi_m1, ovi_m2, ovi_m3, ovi_m4)

Data: dat2
Models:
ovi_m1: keb ~ 1 + (tod2_1 + tod2_3) * (phase + tc1 + wcdt1 + c1 + c2 + c3 + c
4 + game + dalg) + (c1 + c2 + c3 + c4) * tc1 + (1 | Player)
ovi_m2: keb ~ 1 + (tod2_1 + tod2_3) * (phase + tc1 + tc2 + wcdt1 + wcdt2 + c1
+ c2 + c3 + c4 + game + dalg) + (c1 + c2 + c3 + c4) * tc1 + (1 | Player)
ovi_m3: keb ~ 1 + (tod2_1 + tod2_3) * (phase + tc1 + tc2 + wcdt1 + wcdt2 + c1
+ c2 + c3 + c4 + game + dalg) + (c1 + c2 + c3 + c4) * (tc1 + tc2) + (1 | Play
er)
ovi_m4: keb ~ 1 + (tod2_1 + tod2_3) * (phase + tc1 + tc2 + wcdt1 + wcdt2 + c1
+ c2 + c3 + c4 + game + dalg) + (c1 + c2 + c3 + c4) * (tc1 + tc2 + wcdt1 + wc
dt2) + (1 | Player)
       npar   AIC   BIC logLik -2*log(L)  Chisq Df Pr(>Chisq)
ovi_m1   36 25026 25278 -12477     24954
ovi_m2   42 24974 25268 -12445     24890 63.825  6  7.493e-12 ***
ovi_m3   46 24961 25282 -12435     24869 21.406  4  0.0002631 ***
ovi_m4   54 24972 25349 -12432     24864  5.135  8  0.7430583
---
Signif. codes:  0 '***' 0.001 '**' 0.01 '*' 0.05 '.' 0.1 ' ' 1

print(summary(ovi_m2),corr=FALSE)

Linear mixed model fit by maximum likelihood  ['lmerMod']
Formula: keb ~ 1 + (tod2_1 + tod2_3) * (phase + tc1 + tc2 + wcdt1 + wcdt2 +
    c1 + c2 + c3 + c4 + game + dalg) + (c1 + c2 + c3 + c4) *
    tc1 + (1 | Player)
   Data: dat2
Control: lmerControl(calc.derivs = FALSE)

      AIC       BIC    logLik -2*log(L)  df.resid
  24974.5   25267.5  -12445.3   24890.5      7866

Scaled residuals:
    Min      1Q  Median      3Q     Max
-4.3620 -0.6075  0.0769  0.7085  2.6518

Random effects:
 Groups   Name        Variance Std.Dev.
 Player   (Intercept) 0.2784   0.5277
 Residual             1.3478   1.1609
Number of obs: 7908, groups:  Player, 20

Fixed effects:
              Estimate Std. Error t value
(Intercept)   3.765106   0.126146  29.847
tod2_1        0.725388   0.110364   6.573
tod2_3        0.352976   0.110931   3.182
```

```
phase         0.146678   0.044621   3.287
tc1           0.054470   0.013892   3.921
tc2           0.049791   0.007690   6.475
wcdt1        -0.033356   0.013089  -2.548
wcdt2         0.011936   0.006889   1.733
c1            0.897428   0.077176  11.628
c2            0.952950   0.047261  20.163
c3            0.779836   0.044560  17.501
c4            0.006552   0.054576   0.120
game         -0.066858   0.063911  -1.046
dalg         -0.054816   0.073077  -0.750
tod2_1:phase  0.256814   0.110362   2.327
tod2_1:tc1    0.022624   0.038238   0.592
tod2_1:tc2   -0.027780   0.021418  -1.297
tod2_1:wcdt1  0.040738   0.034824   1.170
tod2_1:wcdt2 -0.056279   0.016927  -3.325
tod2_1:c1     0.679744   0.205034   3.315
tod2_1:c2    -0.380631   0.125555  -3.032
tod2_1:c3    -0.372739   0.118375  -3.149
tod2_1:c4     0.414698   0.144979   2.860
tod2_1:game  -0.141946   0.156432  -0.907
tod2_1:dalg  -0.605157   0.181314  -3.338
tod2_3:phase  0.463569   0.110931   4.179
tod2_3:tc1    0.080944   0.031820   2.544
tod2_3:tc2    0.056886   0.017406   3.268
tod2_3:wcdt1 -0.017455   0.025920  -0.673
tod2_3:wcdt2 -0.044707   0.016812  -2.659
tod2_3:c1     0.084979   0.189154   0.449
tod2_3:c2    -0.658004   0.115836  -5.680
tod2_3:c3     0.350330   0.109215   3.208
tod2_3:c4    -0.452838   0.133765  -3.385
tod2_3:game  -0.543366   0.160705  -3.381
tod2_3:dalg  -0.816281   0.174348  -4.682
tc1:c1       -0.081931   0.061024  -1.343
tc1:c2       -0.099592   0.037370  -2.665
tc1:c3       -0.208620   0.035235  -5.921
tc1:c4       -0.047913   0.043156  -1.110
```

- No evidence for interactions between session and Item within championship
- Prefer LMM `ovi_m2` (BCI) [or `ovi_m3` (ACI)]

## Random-effect structure built on **KEB effects**

There are two options to build the random-effect structure:

1. Use effects, that is analogous to fixed effects (but including only main effects and theoretically motivated interactions)
2. Use scores for eight KEB items; yielding something like an analysis of the subscale validity.

Here we estimate GM and effects, that is analogous to fixed effects (but including only main effects and theoretically motivated interactions). Usually, this is the default.

## Over-complex LMM

This model estimates the upper boundary. It is almost guaranteed to be overparameterized. Fitting the following LMMs takes time. Therefore the data (including contrast definitions, etc) and the fitted LMM objects are stored offline and can be loaded. The `ovi_m2` is also included.

```
# sort of a maximal LMM
cpx_m1 <-
lmer(keb ~ 1 + (tod2_1 + tod2_3)*(phase+ tc1+tc2 + wcdt1+wcdt2 +
           c1+c2+c3+c4 + game+dalg) +
          (c1+c2+c3+c4)*(tc1+tc2) +
          (1 + c1+c2+c3+c4 +
           phase +  tc1 + tc2 + wcdt1 + wcdt2 + tod2_1 + tod2_3 + game + dalg
+
           tod2_1:game + tod2_3:game + tod2_1:dalg +tod2_3:dalg | Player),
     dat=dat2, REML=FALSE, control=lmerControl(calc.derivs = FALSE))
summary(rePCA(cpx_m1)) # overparameterized
VarCorr(cpx_m1)
```

## Zero-correlation parameter LMM

- We suppress CPs for effects, except for SSRS.
- **CPs** for `phase`, `tc1`, `tc2`, `wcdt1`, `wcdt2`, `tod2_1`, `tod2_3`, `game`, `dalg` with `c1` to `c4` are not supported.

```
zcp_m1 <-
lmer(keb ~ 1 + (tod2_1 + tod2_3)*(phase+ tc1+tc2 + wcdt1+wcdt2 +
           c1+c2+c3+c4 + game+dalg) + (c1+c2+c3+c4)*(tc1+tc2) +
           (1 + c1+c2+c3+c4 + game + dalg | Player) +
           (0 + tc1+tc2 + phase  + wcdt1+wcdt2 + tod2_1+tod2_3 +
           tod2_1:game + tod2_3:game + tod2_1:dalg +tod2_3:dalg || Player),
      dat=dat2, REML=FALSE, control=lmerControl(calc.derivs = FALSE))
summary(rePCA(zcp_m1)) # supported!
VarCorr(zcp_m1)

# remove item x tc2
zcp_m2 <-
lmer(keb ~ 1 + (tod2_1 + tod2_3)*(phase+ tc1+tc2 + wcdt1+wcdt2 +
           c1+c2+c3+c4 + game+dalg) + tc1:(c1+c2+c3+c4) +
           (1 + c1+c2+c3+c4 | Player) +
           (0 + phase +  tc1 + tc2 + wcdt1 + wcdt2 + tod2_1 + tod2_3 + game +
dalg +
           tod2_1:game + tod2_3:game + tod2_1:dalg +tod2_3:dalg || Player),
     dat=dat2, REML=FALSE, control=lmerControl(calc.derivs = FALSE))
summary(rePCA(zcp_m2)) # supported!
VarCorr(zcp_m2)
anova(zcp_m2x, zcp_m2)
```

```
anova(ovi_m2, zcp_m2, zcp_m1, cpx_m1)

Data: dat2
Models:
ovi_m2: keb ~ 1 + (tod2_1 + tod2_3) * (phase + tc1 + tc2 + wcdt1 + wcdt2 + c1
+ c2 + c3 + c4 + game + dalg) + (c1 + c2 + c3 + c4) * tc1 + (1 | Player)
zcp_m2: keb ~ 1 + (tod2_1 + tod2_3) * (phase + tc1 + tc2 + wcdt1 + wcdt2 + c1
+ c2 + c3 + c4 + game + dalg) + (c1 + c2 + c3 + c4) * tc1 + (1 + c1 + c2 + c3
+ c4 | Player) + ((0 + phase | Player) + (0 + tc1 | Player) + (0 + tc2 | Play
er) + (0 + wcdt1 | Player) + (0 + wcdt2 | Player) + (0 + tod2_1 | Player) + (
0 + tod2_3 | Player) + (0 + game | Player) + (0 + dalg | Player) + (0 + tod2_
1:game | Player) + (0 + tod2_3:game | Player) + (0 + tod2_1:dalg | Player) +
(0 + tod2_3:dalg | Player))
zcp_m1: keb ~ 1 + (tod2_1 + tod2_3) * (phase + tc1 + tc2 + wcdt1 + wcdt2 + c1
+ c2 + c3 + c4 + game + dalg) + (c1 + c2 + c3 + c4) * (tc1 + tc2) + (1 + c1 +
c2 + c3 + c4 | Player) + ((0 + phase | Player) + (0 + tc1 | Player) + (0 + tc
2 | Player) + (0 + wcdt1 | Player) + (0 + wcdt2 | Player) + (0 + tod2_1 | Pla
yer) + (0 + tod2_3 | Player) + (0 + game | Player) + (0 + dalg | Player) + (0
+ tod2_1:game | Player) + (0 + tod2_3:game | Player) + (0 + tod2_1:dalg | Pla
yer) + (0 + tod2_3:dalg | Player))
cpx_m1: keb ~ 1 + (tod2_1 + tod2_3) * (phase + tc1 + tc2 + wcdt1 + wcdt2 + c1
+ c2 + c3 + c4 + game + dalg) + (c1 + c2 + c3 + c4) * (tc1 + tc2) + (1 + c1 +
c2 + c3 + c4 + phase + tc1 + tc2 + wcdt1 + wcdt2 + tod2_1 + tod2_3 + game + d
alg + tod2_1:game + tod2_3:game + tod2_1:dalg + tod2_3:dalg | Player)
       npar   AIC   BIC logLik -2*log(L)  Chisq  Df Pr(>Chisq)
ovi_m2   42 24974 25268 -12445     24890
zcp_m2   69 24047 24528 -11954     23909 981.69  27  < 2.2e-16 ***
zcp_m1   73 24028 24538 -11941     23882  26.53   4  2.474e-05 ***
cpx_m1  216 24063 25569 -11815     23631 251.73 143  5.306e-08 ***
---
Signif. codes:  0 '***' 0.001 '**' 0.01 '*' 0.05 '.' 0.1 ' ' 1
```

- BIC suggests zcp_m2.

## CP-Exploration: Parsimonious LMM

We start with a complex prm_m1; ideally, it should fit as well as cpx_m1.

*Rationale*

- We know that CPs for c1 to c4 with time-related VCs are not supported
- prm_m1: check whether CPs between time-related VCs are supported; answer: BIC says no.
- prm_m2: check whether CPs between time-related VCs are supported; answer: BIC says no.

```
prm_m1 <-
lmer(keb ~  1 + (tod2_1 + tod2_3)*(phase+ tc1+tc2 + wcdt1+wcdt2 +
            c1+c2+c3+c4 + game+dalg) +
            (c1+c2+c3+c4)*tc1 +
            (1 + c1+c2+c3+c4 | Player)  +
            (0 + tc1+tc2 + wcdt1+wcdt2 + tod2_1 + tod2_3 + game + dalg | Play
```

```
er) +
            (0 + tod2_1:game + tod2_3:game + tod2_1:dalg + tod2_3:dalg || Pla
yer),
      dat=dat2, REML=FALSE, control=lmerControl(calc.derivs = FALSE))
summary(rePCA(prm_m1)) # not supported
VarCorr(prm_m1)
anova(zcp_m2, prm_m1, cpx_m1 )

# keep only "within-factor" CPs
prm_m2 <-
lmer(keb ~ 1 + (tod2_1 + tod2_3)*(phase + tc1+tc2 + wcdt1+wcdt2 +
           c1+c2+c3+c4 + game+dalg) +
          (c1+c2+c3+c4)*tc1 +
          (1 + c1+c2+c3+c4 | Player) + (0 + tc1+tc2 + wcdt1+wcdt2 | Player) +
          (0 + tod2_1+tod2_3 | Player) + (0 + game+dalg | Player) +
          (0 + tod2_1:game + tod2_3:game + tod2_1:dalg + tod2_3:dalg || Playe
r),
      dat=dat2, REML=FALSE, control=lmerControl(calc.derivs = FALSE))
summary(rePCA(prm_m2))
VarCorr(prm_m2)
anova(prm_m2, prm_m1)
anova(ovi_m2, zcp_m2, prm_m2, cpx_m1)

# check corr
prm_m3 <-
lmer(keb ~ 1 + (tod2_1 + tod2_3)*(phase + tc1+tc2 + wcdt1+wcdt2 +
           c1+c2+c3+c4 + game+dalg) +
           (c1+c2+c3+c4)*tc1 +
           (1 + c1+c2+c3+c4 | Player) + (0 + tc1+tc2 + wcdt1+wcdt2 | Player)
+
           (0 + tod2_1+tod2_3 + game+dalg +
           tod2_1:game + tod2_3:game + tod2_1:dalg + tod2_3:dalg || Player),
     dat=dat2, REML=FALSE, control=lmerControl(calc.derivs = FALSE))
summary(rePCA(prm_m3))
VarCorr(prm_m3)

#save(ovi_m2, zcp_m1, zcp_m2, prm_m1, prm_m2, prm_m3, cpx_m1,
#      file="./fits/LMMs_JWM2019_v3.rda")

anova(prm_m3, prm_m1)

Data: dat2
Models:
prm_m3: keb ~ 1 + (tod2_1 + tod2_3) * (phase + tc1 + tc2 + wcdt1 + wcdt2 + c1
+ c2 + c3 + c4 + game + dalg) + (c1 + c2 + c3 + c4) * tc1 + (1 + c1 + c2 + c3
+ c4 | Player) + (0 + tc1 + tc2 + wcdt1 + wcdt2 | Player) + ((0 + tod2_1 | Pl
ayer) + (0 + tod2_3 | Player) + (0 + game | Player) + (0 + dalg | Player) + (
0 + tod2_1:game | Player) + (0 + tod2_3:game | Player) + (0 + tod2_1:dalg | P
layer) + (0 + tod2_3:dalg | Player))
prm_m1: keb ~ 1 + (tod2_1 + tod2_3) * (phase + tc1 + tc2 + wcdt1 + wcdt2 + c1
+ c2 + c3 + c4 + game + dalg) + (c1 + c2 + c3 + c4) * tc1 + (1 + c1 + c2 + c3
```

```
+ c4 | Player) + (0 + tc1 + tc2 + wcdt1 + wcdt2 + tod2_1 + tod2_3 + game + da
lg | Player) + ((0 + tod2_1:game | Player) + (0 + game:tod2_3 | Player) + (0
+ tod2_1:dalg | Player) + (0 + tod2_3:dalg | Player))
       npar   AIC   BIC logLik -2*log(L)  Chisq Df Pr(>Chisq)
prm_m3   74 24049 24565 -11950     23901
prm_m1   96 24024 24694 -11916     23832 68.578 22  1.105e-06 ***
---
Signif. codes:  0 '***' 0.001 '**' 0.01 '*' 0.05 '.' 0.1 ' ' 1
```

```
anova(ovi_m2, zcp_m2,  prm_m3, prm_m2, prm_m1, cpx_m1)
```

```
Data: dat2
Models:
ovi_m2: keb ~ 1 + (tod2_1 + tod2_3) * (phase + tc1 + tc2 + wcdt1 + wcdt2 + c1
+ c2 + c3 + c4 + game + dalg) + (c1 + c2 + c3 + c4) * tc1 + (1 | Player)
zcp_m2: keb ~ 1 + (tod2_1 + tod2_3) * (phase + tc1 + tc2 + wcdt1 + wcdt2 + c1
+ c2 + c3 + c4 + game + dalg) + (c1 + c2 + c3 + c4) * tc1 + (1 + c1 + c2 + c3
+ c4 | Player) + ((0 + phase | Player) + (0 + tc1 | Player) + (0 + tc2 | Play
er) + (0 + wcdt1 | Player) + (0 + wcdt2 | Player) + (0 + tod2_1 | Player) + (
0 + tod2_3 | Player) + (0 + game | Player) + (0 + dalg | Player) + (0 + tod2_
1:game | Player) + (0 + tod2_3:game | Player) + (0 + tod2_1:dalg | Player) +
(0 + tod2_3:dalg | Player))
prm_m3: keb ~ 1 + (tod2_1 + tod2_3) * (phase + tc1 + tc2 + wcdt1 + wcdt2 + c1
+ c2 + c3 + c4 + game + dalg) + (c1 + c2 + c3 + c4) * tc1 + (1 + c1 + c2 + c3
+ c4 | Player) + (0 + tc1 + tc2 + wcdt1 + wcdt2 | Player) + ((0 + tod2_1 | Pl
ayer) + (0 + tod2_3 | Player) + (0 + game | Player) + (0 + dalg | Player) + (
0 + tod2_1:game | Player) + (0 + tod2_3:game | Player) + (0 + tod2_1:dalg | P
layer) + (0 + tod2_3:dalg | Player))
prm_m2: keb ~ 1 + (tod2_1 + tod2_3) * (phase + tc1 + tc2 + wcdt1 + wcdt2 + c1
+ c2 + c3 + c4 + game + dalg) + (c1 + c2 + c3 + c4) * tc1 + (1 + c1 + c2 + c3
+ c4 | Player) + (0 + tc1 + tc2 + wcdt1 + wcdt2 | Player) + (0 + tod2_1 + tod
2_3 | Player) + (0 + game + dalg | Player) + ((0 + tod2_1:game | Player) + (0
+ game:tod2_3 | Player) + (0 + tod2_1:dalg | Player) + (0 + tod2_3:dalg | Pla
yer))
prm_m1: keb ~ 1 + (tod2_1 + tod2_3) * (phase + tc1 + tc2 + wcdt1 + wcdt2 + c1
+ c2 + c3 + c4 + game + dalg) + (c1 + c2 + c3 + c4) * tc1 + (1 + c1 + c2 + c3
+ c4 | Player) + (0 + tc1 + tc2 + wcdt1 + wcdt2 + tod2_1 + tod2_3 + game + da
lg | Player) + ((0 + tod2_1:game | Player) + (0 + game:tod2_3 | Player) + (0
+ tod2_1:dalg | Player) + (0 + tod2_3:dalg | Player))
cpx_m1: keb ~ 1 + (tod2_1 + tod2_3) * (phase + tc1 + tc2 + wcdt1 + wcdt2 + c1
+ c2 + c3 + c4 + game + dalg) + (c1 + c2 + c3 + c4) * (tc1 + tc2) + (1 + c1 +
c2 + c3 + c4 + phase + tc1 + tc2 + wcdt1 + wcdt2 + tod2_1 + tod2_3 + game + d
alg + tod2_1:game + tod2_3:game + tod2_1:dalg + tod2_3:dalg | Player)
       npar   AIC   BIC logLik -2*log(L)    Chisq  Df Pr(>Chisq)
ovi_m2   42 24974 25268 -12445     24890
zcp_m2   69 24047 24528 -11954     23909 981.6925  27  < 2.2e-16 ***
prm_m3   74 24049 24565 -11950     23901   8.2539   5   0.142788
prm_m2   76 24029 24559 -11938     23877  24.0188   2  6.087e-06 ***
prm_m1   96 24024 24694 -11916     23832  44.5590  20   0.001266 **
cpx_m1  216 24063 25569 -11815     23631 201.4310 120  4.679e-06 ***
---
Signif. codes:  0 '***' 0.001 '**' 0.01 '*' 0.05 '.' 0.1 ' ' 1
```

```
model_parameters(zcp_m2, dv.labels = c("zcp_m2"))

# Fixed Effects

Parameter       | Coefficient |       SE |         95% CI | t(7839) |      p
---------------------------------------------------------------------------
(Intercept)     |        3.76 |     0.13 | [ 3.50,  4.02] |   28.17 | < .001
tod2 1          |        0.74 |     0.12 | [ 0.50,  0.98] |    5.97 | < .001
tod2 3          |        0.37 |     0.11 | [ 0.15,  0.59] |    3.30 | < .001
phase           |        0.14 |     0.05 | [ 0.04,  0.24] |    2.66 | 0.008
tc1             |        0.05 |     0.03 | [ 0.00,  0.10] |    2.03 | 0.043
tc2             |        0.05 |     0.01 | [ 0.02,  0.08] |    3.64 | < .001
wcdt1           |       -0.04 |     0.02 | [-0.07,  0.00] |   -1.78 | 0.075
wcdt2           |        0.01 | 8.82e-03 | [-0.01,  0.03] |    1.38 | 0.167
c1              |        0.90 |     0.16 | [ 0.58,  1.22] |    5.54 | < .001
c2              |        0.95 |     0.10 | [ 0.77,  1.14] |    9.95 | < .001
c3              |        0.78 |     0.16 | [ 0.46,  1.10] |    4.82 | < .001
c4              |    8.03e-03 |     0.11 | [-0.21,  0.23] |    0.07 | 0.944
game            |       -0.06 |     0.08 | [-0.20,  0.09] |   -0.74 | 0.462
dalg            |       -0.04 |     0.08 | [-0.20,  0.13] |   -0.46 | 0.648
tod2 1 × phase  |        0.27 |     0.10 | [ 0.07,  0.47] |    2.70 | 0.007
tod2 1 × tc1    |        0.01 |     0.03 | [-0.06,  0.08] |    0.38 | 0.703
tod2 1 × tc2    |       -0.02 |     0.02 | [-0.06,  0.01] |   -1.20 | 0.231
tod2 1 × wcdt1  |        0.05 |     0.03 | [-0.02,  0.11] |    1.42 | 0.155
tod2 1 × wcdt2  |       -0.06 |     0.02 | [-0.09, -0.03] |   -3.71 | < .001
tod2 1 × c1     |        0.68 |     0.19 | [ 0.31,  1.04] |    3.62 | < .001
tod2 1 × c2     |       -0.38 |     0.11 | [-0.60, -0.15] |   -3.29 | 0.001
tod2 1 × c3     |       -0.37 |     0.11 | [-0.58, -0.16] |   -3.43 | < .001
tod2 1 × c4     |        0.42 |     0.13 | [ 0.16,  0.68] |    3.17 | 0.002
tod2 1 × game   |       -0.16 |     0.17 | [-0.48,  0.17] |   -0.94 | 0.347
tod2 1 × dalg   |       -0.63 |     0.18 | [-0.99, -0.28] |   -3.51 | < .001
tod2 3 × phase  |        0.48 |     0.10 | [ 0.28,  0.68] |    4.76 | < .001
tod2 3 × tc1    |        0.07 |     0.03 | [ 0.02,  0.13] |    2.54 | 0.011
tod2 3 × tc2    |        0.06 |     0.02 | [ 0.03,  0.09] |    3.72 | < .001
tod2 3 × wcdt1  |       -0.02 |     0.02 | [-0.06,  0.03] |   -0.71 | 0.480
tod2 3 × wcdt2  |       -0.04 |     0.02 | [-0.07, -0.01] |   -2.90 | 0.004
tod2 3 × c1     |        0.09 |     0.17 | [-0.25,  0.43] |    0.53 | 0.596
tod2 3 × c2     |       -0.65 |     0.11 | [-0.86, -0.44] |   -6.17 | < .001
tod2 3 × c3     |        0.36 |     0.10 | [ 0.16,  0.55] |    3.61 | < .001
tod2 3 × c4     |       -0.45 |     0.12 | [-0.69, -0.21] |   -3.66 | < .001
tod2 3 × game   |       -0.59 |     0.18 | [-0.93, -0.24] |   -3.34 | < .001
tod2 3 × dalg   |       -0.86 |     0.19 | [-1.22, -0.49] |   -4.61 | < .001
tc1 × c1        |       -0.08 |     0.06 | [-0.19,  0.03] |   -1.49 | 0.137
tc1 × c2        |       -0.10 |     0.03 | [-0.17, -0.04] |   -2.99 | 0.003
tc1 × c3        |       -0.21 |     0.03 | [-0.27, -0.15] |   -6.49 | < .001
tc1 × c4        |       -0.05 |     0.04 | [-0.12,  0.03] |   -1.20 | 0.229

# Random Effects

Parameter                   | Coefficient
-----------------------------------------
```

```
SD (Intercept: Player)      |         0.57
SD (c1: Player)             |         0.65
SD (c2: Player)             |         0.38
SD (c3: Player)             |         0.70
SD (c4: Player)             |         0.46
SD (phase: Player)          |         0.15
SD (tc1: Player)            |         0.10
SD (tc2: Player)            |         0.05
SD (wcdt1: Player)          |         0.07
SD (wcdt2: Player)          |         0.03
SD (tod2_1: Player)         |         0.32
SD (tod2_3: Player)         |         0.22
SD (game: Player)           |         0.21
SD (dalg: Player)           |         0.23
SD (tod2_1:game: Player)    |         0.37
SD (tod2_3:game: Player)    |         0.42
SD (tod2_1:dalg: Player)    |         0.32
SD (tod2_3:dalg: Player)    |         0.43
Cor (Intercept~c1: Player) |         0.19
Cor (Intercept~c2: Player) |         0.11
Cor (Intercept~c3: Player) |        -0.11
Cor (Intercept~c4: Player) |        -0.45
Cor (c1~c2: Player)         |         0.15
Cor (c1~c3: Player)         |         0.19
Cor (c1~c4: Player)         |         0.42
Cor (c2~c3: Player)         |         0.46
Cor (c2~c4: Player)         |         0.48
Cor (c3~c4: Player)         |         0.70
SD (Residual)               |         1.06


Uncertainty intervals (equal-tailed) and p-values (two-tailed) computed
  using a Wald t-distribution approximation. Uncertainty intervals for
  random effect variances computed using a Wald z-distribution
  approximation.
```

- AIC and BIC select `zcp_m2`
- Just not enough support for CPs given the small sample.

The selection depends on whether we view it from a confirmatory or exploratory perspective. Also note that there are quite a few CPs in `zcp_m2`. We vote for `zcp_m2` for inferential purposes. We may still want to look at CPs in `prm_m3` for heuristic purposes.

# Fixed-effect explorations

## LMM `zcp_m3` and LMM `zcp_m4`

LMM `zcp_m2` extended with three-variable fixed-effect interactions.

```
zcp_m3 <-
lmer(keb ~ 1 + (tod2_1 + tod2_3)*((phase + tc1+tc2 +
           wcdt1+wcdt2)*(c1+c2+c3+c4) + game+dalg) +
           (c1+c2+c3+c4)*tc1 +
           (1 + c1+c2+c3+c4 | Player) +
           (0 + phase +  tc1 + tc2 + wcdt1 + wcdt2 + tod2_1 + tod2_3 + game +
dalg +
           tod2_1:game + tod2_3:game + tod2_1:dalg +tod2_3:dalg || Player),
     dat=dat2, REML=FALSE, control=lmerControl(calc.derivs = FALSE))
summary(rePCA(zcp_m3)) # supported!

$Player
Importance of components:
                         [,1]   [,2]   [,3]   [,4]    [,5]    [,6]   [,7]
Standard deviation     0.8179 0.6359 0.5120 0.4119 0.40416 0.35374 0.3141
Proportion of Variance 0.2949 0.1782 0.1156 0.0748 0.07201 0.05516 0.0435
Cumulative Proportion  0.2949 0.4731 0.5887 0.6635 0.73549 0.79065 0.8341
                          [,8]    [,9]   [,10]   [,11]   [,12]  [,13]   [,14]
Standard deviation     0.30506 0.30456 0.21572 0.21394 0.20100 0.1460 0.13979
Proportion of Variance 0.04102 0.04089 0.02051 0.02018 0.01781 0.0094 0.00861
Cumulative Proportion  0.87517 0.91607 0.93658 0.95676 0.97457 0.9840 0.99258
                         [,15]   [,16]   [,17]   [,18]
Standard deviation     0.09575 0.06677 0.04999 0.02662
Proportion of Variance 0.00404 0.00197 0.00110 0.00031
Cumulative Proportion  0.99662 0.99859 0.99969 1.00000

VarCorr(zcp_m3)

 Groups    Name        Std.Dev. Corr
 Player    (Intercept) 0.569207
           c1          0.655013  0.194
           c2          0.383556  0.105  0.149
           c3          0.705262 -0.113  0.187  0.459
           c4          0.457808 -0.451  0.421  0.483  0.697
 Player.1  phase       0.146893
 Player.2  tc1         0.100618
 Player.3  tc2         0.052527
 Player.4  wcdt1       0.070161
 Player.5  wcdt2       0.027970
 Player.6  tod2_1      0.320568
 Player.7  tod2_3      0.224811
 Player.8  game        0.211220
 Player.9  dalg        0.226689
 Player.10 tod2_1:game 0.371722
 Player.11 tod2_3:game 0.424703
 Player.12 tod2_1:dalg 0.320047
 Player.13 tod2_3:dalg 0.432854
 Residual              1.050835

anova(zcp_m2, zcp_m3)
```

```
Data: dat2
Models:
zcp_m2: keb ~ 1 + (tod2_1 + tod2_3) * (phase + tc1 + tc2 + wcdt1 + wcdt2 + c1
+ c2 + c3 + c4 + game + dalg) + (c1 + c2 + c3 + c4) * tc1 + (1 + c1 + c2 + c3
+ c4 | Player) + ((0 + phase | Player) + (0 + tc1 | Player) + (0 + tc2 | Play
er) + (0 + wcdt1 | Player) + (0 + wcdt2 | Player) + (0 + tod2_1 | Player) + (
0 + tod2_3 | Player) + (0 + game | Player) + (0 + dalg | Player) + (0 + tod2_
1:game | Player) + (0 + tod2_3:game | Player) + (0 + tod2_1:dalg | Player) +
(0 + tod2_3:dalg | Player))
zcp_m3: keb ~ 1 + (tod2_1 + tod2_3) * ((phase + tc1 + tc2 + wcdt1 + wcdt2) *
(c1 + c2 + c3 + c4) + game + dalg) + (c1 + c2 + c3 + c4) * tc1 + (1 + c1 + c2
+ c3 + c4 | Player) + ((0 + phase | Player) + (0 + tc1 | Player) + (0 + tc2 |
Player) + (0 + wcdt1 | Player) + (0 + wcdt2 | Player) + (0 + tod2_1 | Player)
+ (0 + tod2_3 | Player) + (0 + game | Player) + (0 + dalg | Player) + (0 + to
d2_1:game | Player) + (0 + tod2_3:game | Player) + (0 + tod2_1:dalg | Player)
+ (0 + tod2_3:dalg | Player))
       npar   AIC   BIC logLik -2*log(L)  Chisq Df Pr(>Chisq)
zcp_m2   69 24047 24528 -11954     23909
zcp_m3  125 24070 24942 -11910     23820 88.684 56   0.003523 **
---
Signif. codes:  0 '***' 0.001 '**' 0.01 '*' 0.05 '.' 0.1 ' ' 1
```

```
compare_parameters(zcp_m2, zcp_m3)
```

```
Parameter                |                   zcp_m2 |                   zcp_m3
------------------------------------------------------------------------------
(Intercept)              |     3.76 ( 3.50,  4.02) |     3.76 ( 3.50,  4.02)
tod2 1                   |     0.74 ( 0.50,  0.98) |     0.74 ( 0.50,  0.98)
tod2 3                   |     0.37 ( 0.15,  0.59) |     0.37 ( 0.15,  0.59)
phase                    |     0.14 ( 0.04,  0.24) |     0.14 ( 0.04,  0.24)
tc1                      |     0.05 ( 0.00,  0.10) |     0.05 ( 0.00,  0.10)
tc2                      |     0.05 ( 0.02,  0.08) |     0.05 ( 0.02,  0.08)
wcdt1                    |    -0.04 (-0.07,  0.00) |    -0.04 (-0.07,  0.00)
wcdt2                    |     0.01 (-0.01,  0.03) |     0.01 (-0.01,  0.03)
c1                       |     0.90 ( 0.58,  1.22) |     0.95 ( 0.60,  1.31)
c2                       |     0.95 ( 0.77,  1.14) |     0.99 ( 0.78,  1.20)
c3                       |     0.78 ( 0.46,  1.10) |     0.72 ( 0.39,  1.05)
c4                       | 8.03e-03 (-0.21,  0.23) |     0.08 (-0.17,  0.33)
game                     |    -0.06 (-0.20,  0.09) |    -0.06 (-0.20,  0.09)
dalg                     |    -0.04 (-0.20,  0.13) |    -0.04 (-0.20,  0.13)
tod2 1 × phase           |     0.27 ( 0.07,  0.47) |     0.27 ( 0.08,  0.47)
tod2 1 × tc1             |     0.01 (-0.06,  0.08) |     0.01 (-0.05,  0.08)
tod2 1 × tc2             |    -0.02 (-0.06,  0.01) |    -0.02 (-0.06,  0.01)
tod2 1 × wcdt1           |     0.05 (-0.02,  0.11) |     0.05 (-0.02,  0.11)
tod2 1 × wcdt2           |    -0.06 (-0.09, -0.03) |    -0.06 (-0.09, -0.03)
tod2 1 × c1              |     0.68 ( 0.31,  1.04) |     0.58 ( 0.03,  1.13)
tod2 1 × c2              |    -0.38 (-0.60, -0.15) |    -0.28 (-0.62,  0.06)
tod2 1 × c3              |    -0.37 (-0.58, -0.16) |    -0.40 (-0.71, -0.08)
tod2 1 × c4              |     0.42 ( 0.16,  0.68) |     0.43 ( 0.04,  0.82)
tod2 1 × game            |    -0.16 (-0.48,  0.17) |    -0.16 (-0.48,  0.17)
tod2 1 × dalg            |    -0.63 (-0.99, -0.28) |    -0.63 (-0.99, -0.28)
tod2 3 × phase           |     0.48 ( 0.28,  0.68) |     0.48 ( 0.29,  0.68)
```

| | | |
|---|---|---|
| tod2 3 × tc1 | 0.07 ( 0.02, 0.13) | 0.07 ( 0.02, 0.13) |
| tod2 3 × tc2 | 0.06 ( 0.03, 0.09) | 0.06 ( 0.03, 0.09) |
| tod2 3 × wcdt1 | -0.02 (-0.06, 0.03) | -0.02 (-0.06, 0.03) |
| tod2 3 × wcdt2 | -0.04 (-0.07, -0.01) | -0.04 (-0.07, -0.01) |
| tod2 3 × c1 | 0.09 (-0.25, 0.43) | 0.06 (-0.44, 0.56) |
| tod2 3 × c2 | -0.65 (-0.86, -0.44) | -0.35 (-0.66, -0.05) |
| tod2 3 × c3 | 0.36 ( 0.16, 0.55) | 0.67 ( 0.38, 0.96) |
| tod2 3 × c4 | -0.45 (-0.69, -0.21) | -0.48 (-0.83, -0.13) |
| tod2 3 × game | -0.59 (-0.93, -0.24) | -0.59 (-0.93, -0.24) |
| tod2 3 × dalg | -0.86 (-1.22, -0.49) | -0.86 (-1.22, -0.49) |
| tc1 × c1 | -0.08 (-0.19, 0.03) | -0.08 (-0.22, 0.06) |
| tc1 × c2 | -0.10 (-0.17, -0.04) | -0.04 (-0.13, 0.04) |
| tc1 × c3 | -0.21 (-0.27, -0.15) | -0.21 (-0.29, -0.13) |
| tc1 × c4 | -0.05 (-0.12, 0.03) | -0.04 (-0.14, 0.06) |
| wcdt1 × c1 | | 0.01 (-0.09, 0.11) |
| phase × c1 | | 0.03 (-0.18, 0.24) |
| phase × c2 | | -0.04 (-0.17, 0.09) |
| phase × c3 | | -0.18 (-0.30, -0.06) |
| phase × c4 | | -0.06 (-0.21, 0.08) |
| wcdt2 × c2 | | -9.56e-03 (-0.04, 0.03) |
| wcdt2 × c3 | | 0.02 (-0.02, 0.05) |
| wcdt2 × c4 | | -3.33e-03 (-0.04, 0.04) |
| (tod2 1 × tc1) × c1 | | -0.23 (-0.62, 0.15) |
| tc2 × c1 | | -0.04 (-0.12, 0.04) |
| tc2 × c2 | | -0.02 (-0.07, 0.02) |
| tc2 × c3 | | 0.03 (-0.02, 0.07) |
| tc2 × c4 | | -0.03 (-0.09, 0.02) |
| (tod2 1 × tc2) × c2 | | -0.02 (-0.15, 0.11) |
| wcdt1 × c2 | | 0.02 (-0.04, 0.08) |
| wcdt1 × c3 | | -7.64e-03 (-0.07, 0.05) |
| wcdt1 × c4 | | -7.71e-03 (-0.08, 0.06) |
| wcdt2 × c1 | | 0.01 (-0.04, 0.07) |
| (tod2 1 × phase) × c2 | | 0.09 (-0.25, 0.43) |
| (tod2 1 × phase) × c3 | | 0.32 ( 0.00, 0.63) |
| (tod2 1 × phase) × c4 | | -0.31 (-0.70, 0.08) |
| (tod2 1 × wcdt2) × c2 | | -0.05 (-0.14, 0.04) |
| (tod2 1 × tc1) × c2 | | 0.15 (-0.08, 0.39) |
| (tod2 1 × tc1) × c3 | | 0.13 (-0.09, 0.35) |
| (tod2 1 × tc1) × c4 | | -6.02e-03 (-0.28, 0.27) |
| (tod2 1 × tc2) × c1 | | 0.04 (-0.17, 0.26) |
| (tod2 3 × phase) × c3 | | -0.13 (-0.41, 0.16) |
| (tod2 1 × tc2) × c3 | | 0.03 (-0.10, 0.15) |
| (tod2 1 × tc2) × c4 | | -0.03 (-0.18, 0.12) |
| (tod2 1 × wcdt1) × c1 | | 0.01 (-0.23, 0.26) |
| (tod2 1 × phase) × c1 | | -0.27 (-0.82, 0.28) |
| (tod2 1 × wcdt1) × c3 | | 9.96e-04 (-0.14, 0.14) |
| (tod2 1 × wcdt1) × c4 | | -0.02 (-0.19, 0.16) |
| (tod2 1 × wcdt2) × c1 | | 0.05 (-0.09, 0.19) |
| (tod2 3 × tc2) × c3 | | -0.11 (-0.21, -0.01) |
| (tod2 1 × wcdt2) × c3 | | -0.03 (-0.11, 0.05) |
| (tod2 1 × wcdt2) × c4 | | 0.03 (-0.07, 0.13) |

```
(tod2 3 × phase) × c1 |                           |     -0.15 (-0.65,  0.35)
(tod2 3 × phase) × c2 |                           |     -0.01 (-0.32,  0.29)
(tod2 3 × wcdt1) × c4 |                           |      0.03 (-0.14,  0.20)
(tod2 3 × phase) × c4 |                           |     -0.09 (-0.44,  0.26)
(tod2 3 × tc1) × c1   |                           |     -0.22 (-0.54,  0.10)
(tod2 3 × tc1) × c2   |                           |      0.16 (-0.03,  0.36)
(tod2 1 × wcdt1) × c2 |                           |     -0.03 (-0.18,  0.12)
(tod2 3 × tc1) × c4   |                           |     -0.07 (-0.30,  0.16)
(tod2 3 × tc2) × c1   |                           |     -0.03 (-0.21,  0.14)
(tod2 3 × tc2) × c2   |                           |     -0.13 (-0.24, -0.03)
(tod2 3 × wcdt1) × c3 |                           |      0.06 (-0.09,  0.20)
(tod2 3 × tc2) × c4   |                           |  3.80e-03 (-0.12,  0.13)
(tod2 3 × wcdt1) × c1 |                           |      0.08 (-0.17,  0.32)
(tod2 3 × wcdt1) × c2 |                           |      0.01 (-0.14,  0.16)
(tod2 3 × wcdt2) × c3 |                           |     -0.06 (-0.14,  0.02)
(tod2 3 × tc1) × c3   |                           |      0.06 (-0.12,  0.25)
(tod2 3 × wcdt2) × c1 |                           |      0.01 (-0.13,  0.15)
(tod2 3 × wcdt2) × c2 |                           |     -0.05 (-0.14,  0.03)
(tod2 3 × wcdt2) × c4 |                           |      0.01 (-0.09,  0.11)
--------------------------------------------------------------------------
Observations          |                      7908 |                     7908

# potentially interesting: phase:c3

zcp_m4 <-
lmer(keb ~ 1 + (tod2_1 + tod2_3)*(phase + tc1+tc2 +
           wcdt1+wcdt2 + c1+c2+c3+c4 + game+dalg) +
           phase:c3 +
           (c1+c2+c3+c4)*tc1 +
           (1 + c1+c2+c3+c4 | Player) +
           (0 + phase +  tc1 + tc2 + wcdt1 + wcdt2 + tod2_1 + tod2_3 + game +
dalg +
           tod2_1:game + tod2_3:game + tod2_1:dalg +tod2_3:dalg || Player),
     dat=dat2, REML=FALSE, control=lmerControl(calc.derivs = FALSE))
summary(rePCA(zcp_m4)) # supported!

$Player
Importance of components:
                         [,1]   [,2]   [,3]    [,4]    [,5]    [,6]    [,7]
Standard deviation     0.8131 0.6319 0.5085 0.40824 0.40010 0.35227 0.31238
Proportion of Variance 0.2956 0.1785 0.1156 0.07451 0.07157 0.05548 0.04363
Cumulative Proportion  0.2956 0.4741 0.5897 0.66423 0.73580 0.79129 0.83492
                         [,8]    [,9]   [,10]   [,11]   [,12]   [,13]   [,14]
Standard deviation     0.3024 0.30089 0.21468 0.21131 0.19942 0.14438 0.13851
Proportion of Variance 0.0409 0.04048 0.02061 0.01996 0.01778 0.00932 0.00858
Cumulative Proportion  0.8758 0.91629 0.93690 0.95686 0.97464 0.98396 0.99254
                         [,15]   [,16]   [,17]   [,18]
Standard deviation     0.09550 0.06630 0.04978 0.02630
Proportion of Variance 0.00408 0.00197 0.00111 0.00031
Cumulative Proportion  0.99662 0.99858 0.99969 1.00000

VarCorr(zcp_m4)
```

```
 Groups     Name        Std.Dev. Corr
 Player     (Intercept) 0.568956
            c1          0.653799  0.195
            c2          0.383035  0.109  0.148
            c3          0.704151 -0.113  0.190  0.459
            c4          0.456695 -0.450  0.422  0.482  0.698
 Player.1   phase       0.146239
 Player.2   tc1         0.100829
 Player.3   tc2         0.052556
 Player.4   wcdt1       0.070001
 Player.5   wcdt2       0.027772
 Player.6   tod2_1      0.319315
 Player.7   tod2_3      0.223101
 Player.8   game        0.210547
 Player.9   dalg        0.226657
 Player.10 tod2_1:game 0.371920
 Player.11 tod2_3:game 0.422416
 Player.12 tod2_1:dalg 0.317677
 Player.13 tod2_3:dalg 0.431009
 Residual               1.055785
```

```
anova(zcp_m2, zcp_m4)
```

```
Data: dat2
Models:
zcp_m2: keb ~ 1 + (tod2_1 + tod2_3) * (phase + tc1 + tc2 + wcdt1 + wcdt2 + c1
+ c2 + c3 + c4 + game + dalg) + (c1 + c2 + c3 + c4) * tc1 + (1 + c1 + c2 + c3
+ c4 | Player) + ((0 + phase | Player) + (0 + tc1 | Player) + (0 + tc2 | Play
er) + (0 + wcdt1 | Player) + (0 + wcdt2 | Player) + (0 + tod2_1 | Player) + (
0 + tod2_3 | Player) + (0 + game | Player) + (0 + dalg | Player) + (0 + tod2_
1:game | Player) + (0 + tod2_3:game | Player) + (0 + tod2_1:dalg | Player) +
(0 + tod2_3:dalg | Player))
zcp_m4: keb ~ 1 + (tod2_1 + tod2_3) * (phase + tc1 + tc2 + wcdt1 + wcdt2 + c1
+ c2 + c3 + c4 + game + dalg) + phase:c3 + (c1 + c2 + c3 + c4) * tc1 + (1 + c
1 + c2 + c3 + c4 | Player) + ((0 + phase | Player) + (0 + tc1 | Player) + (0
+ tc2 | Player) + (0 + wcdt1 | Player) + (0 + wcdt2 | Player) + (0 + tod2_1 |
Player) + (0 + tod2_3 | Player) + (0 + game | Player) + (0 + dalg | Player) +
(0 + tod2_1:game | Player) + (0 + tod2_3:game | Player) + (0 + tod2_1:dalg |
Player) + (0 + tod2_3:dalg | Player))
       npar   AIC   BIC logLik -2*log(L)  Chisq Df Pr(>Chisq)
zcp_m2   69 24047 24528 -11954     23909
zcp_m4   70 24031 24519 -11945     23891 18.146  1  2.046e-05 ***
---
Signif. codes:  0 '***' 0.001 '**' 0.01 '*' 0.05 '.' 0.1 ' ' 1
```

```
compare_parameters(zcp_m2, zcp_m4)
```

```
Parameter      |                  zcp_m2 |                  zcp_m4
------------------------------------------------------------------
(Intercept)    |     3.76 ( 3.50,  4.02) |     3.76 ( 3.50,  4.02)
tod2 1         |     0.74 ( 0.50,  0.98) |     0.74 ( 0.50,  0.98)
tod2 3         |     0.37 ( 0.15,  0.59) |     0.37 ( 0.15,  0.59)
```

```
phase          |      0.14 ( 0.04,  0.24) |      0.14 ( 0.04,  0.24)
tc1            |      0.05 ( 0.00,  0.10) |      0.05 ( 0.00,  0.10)
tc2            |      0.05 ( 0.02,  0.08) |      0.05 ( 0.02,  0.08)
wcdt1          |     -0.04 (-0.07,  0.00) |     -0.04 (-0.07,  0.00)
wcdt2          |      0.01 (-0.01,  0.03) |      0.01 (-0.01,  0.03)
c1             |      0.90 ( 0.58,  1.22) |      0.90 ( 0.58,  1.22)
c2             |      0.95 ( 0.77,  1.14) |      0.95 ( 0.77,  1.14)
c3             |      0.78 ( 0.46,  1.10) |      0.77 ( 0.45,  1.09)
c4             | 8.03e-03 (-0.21,  0.23) | 7.74e-03 (-0.21,  0.23)
game           |     -0.06 (-0.20,  0.09) |     -0.06 (-0.20,  0.09)
dalg           |     -0.04 (-0.20,  0.13) |     -0.04 (-0.20,  0.13)
tod2 1 × phase |      0.27 ( 0.07,  0.47) |      0.27 ( 0.07,  0.47)
tod2 1 × tc1   |      0.01 (-0.06,  0.08) |      0.01 (-0.06,  0.08)
tod2 1 × tc2   |     -0.02 (-0.06,  0.01) |     -0.02 (-0.06,  0.01)
tod2 1 × wcdt1 |      0.05 (-0.02,  0.11) |      0.05 (-0.02,  0.11)
tod2 1 × wcdt2 |     -0.06 (-0.09, -0.03) |     -0.06 (-0.09, -0.03)
tod2 1 × c1    |      0.68 ( 0.31,  1.04) |      0.68 ( 0.31,  1.04)
tod2 1 × c2    |     -0.38 (-0.60, -0.15) |     -0.38 (-0.60, -0.15)
tod2 1 × c3    |     -0.37 (-0.58, -0.16) |     -0.37 (-0.58, -0.16)
tod2 1 × c4    |      0.42 ( 0.16,  0.68) |      0.42 ( 0.16,  0.68)
tod2 1 × game  |     -0.16 (-0.48,  0.17) |     -0.16 (-0.48,  0.17)
tod2 1 × dalg  |     -0.63 (-0.99, -0.28) |     -0.63 (-0.99, -0.28)
tod2 3 × phase |      0.48 ( 0.28,  0.68) |      0.48 ( 0.28,  0.68)
tod2 3 × tc1   |      0.07 ( 0.02,  0.13) |      0.07 ( 0.02,  0.13)
tod2 3 × tc2   |      0.06 ( 0.03,  0.09) |      0.06 ( 0.03,  0.09)
tod2 3 × wcdt1 |     -0.02 (-0.06,  0.03) |     -0.02 (-0.06,  0.03)
tod2 3 × wcdt2 |     -0.04 (-0.07, -0.01) |     -0.04 (-0.07, -0.01)
tod2 3 × c1    |      0.09 (-0.25,  0.43) |      0.09 (-0.25,  0.43)
tod2 3 × c2    |     -0.65 (-0.86, -0.44) |     -0.65 (-0.86, -0.44)
tod2 3 × c3    |      0.36 ( 0.16,  0.55) |      0.42 ( 0.22,  0.62)
tod2 3 × c4    |     -0.45 (-0.69, -0.21) |     -0.45 (-0.68, -0.21)
tod2 3 × game  |     -0.59 (-0.93, -0.24) |     -0.59 (-0.93, -0.24)
tod2 3 × dalg  |     -0.86 (-1.22, -0.49) |     -0.86 (-1.22, -0.49)
tc1 × c1       |     -0.08 (-0.19,  0.03) |     -0.08 (-0.19,  0.03)
tc1 × c2       |     -0.10 (-0.17, -0.04) |     -0.10 (-0.17, -0.04)
tc1 × c3       |     -0.21 (-0.27, -0.15) |     -0.20 (-0.26, -0.13)
tc1 × c4       |     -0.05 (-0.12,  0.03) |     -0.05 (-0.12,  0.03)
phase × c3     |                          |     -0.17 (-0.25, -0.09)
------------------------------------------------------------------
Observations   |                     7908 |                     7908
```

Only one interaction is of possible interest for follow up: `phase:c3`

## LMM `zcp_m5` and `zcp_m6`

Do we need game in the model? We remove terms from fixed effects and RES.

```
# check fixed effect of game and its interactions with ToD
zcp_m5 <-
lmer(keb ~ 1 + (tod2_1 + tod2_3)*(phase+ tc1+tc2 + wcdt1+wcdt2 +
           c1+c2+c3+c4 + dalg) + tc1:(c1+c2+c3+c4) +
           (1 + c1+c2+c3+c4 | Player) +
```

```
           (0 + phase +  tc1 + tc2 + wcdt1 + wcdt2 + tod2_1 + tod2_3 + game +
dalg +
            tod2_1:game + tod2_3:game + tod2_1:dalg +tod2_3:dalg || Player),
     dat=dat2, REML=FALSE, control=lmerControl(calc.derivs = FALSE))
summary(rePCA(zcp_m5)) # supported!

$Player
Importance of components:
                         [,1]   [,2]   [,3]   [,4]    [,5]    [,6]    [,7]
Standard deviation     0.8117 0.6313 0.5071 0.4816 0.40090 0.34258 0.31166
Proportion of Variance 0.2867 0.1734 0.1119 0.1009 0.06993 0.05107 0.04226
Cumulative Proportion  0.2867 0.4601 0.5719 0.6729 0.74281 0.79388 0.83614
                          [,8]    [,9]   [,10]   [,11]   [,12]   [,13]   [,14
]
Standard deviation     0.30612 0.30336 0.21711 0.21510 0.20244 0.14443 0.1378
7
Proportion of Variance 0.04078 0.04004 0.02051 0.02013 0.01783 0.00908 0.0082
7
Cumulative Proportion  0.87692 0.91696 0.93747 0.95761 0.97544 0.98451 0.9927
8
                         [,15]   [,16]   [,17]   [,18]
Standard deviation     0.09518 0.06609 0.04964 0.02632
Proportion of Variance 0.00394 0.00190 0.00107 0.00030
Cumulative Proportion  0.99673 0.99863 0.99970 1.00000

zcp_m6 <-
lmer(keb ~ 1 + (tod2_1 + tod2_3)*(phase+ tc1+tc2 + wcdt1+wcdt2 +
           c1+c2+c3+c4 + dalg) + tc1:(c1+c2+c3+c4) +
           (1 + c1+c2+c3+c4 | Player) +
           (0 + phase +  tc1 + tc2 + wcdt1 + wcdt2 + tod2_1 + tod2_3 + dalg +
           tod2_1:dalg +tod2_3:dalg || Player),
      dat=dat2, REML=FALSE, control=lmerControl(calc.derivs = FALSE))
summary(rePCA(zcp_m6)) # supported!

$Player
Importance of components:
                         [,1]   [,2]   [,3]    [,4]    [,5]    [,6]    [,7]
Standard deviation     0.8052 0.6520 0.4954 0.39843 0.33305 0.30815 0.29443
Proportion of Variance 0.3407 0.2234 0.1290 0.08341 0.05828 0.04989 0.04555
Cumulative Proportion  0.3407 0.5641 0.6930 0.77644 0.83472 0.88461 0.93016
                          [,8]    [,9]   [,10]   [,11]   [,12]   [,13]   [,14
]
Standard deviation     0.18286 0.17891 0.17082 0.14185 0.09984 0.06848 0.0509
5
Proportion of Variance 0.01757 0.01682 0.01533 0.01057 0.00524 0.00246 0.0013
6
Cumulative Proportion  0.94773 0.96455 0.97988 0.99046 0.99570 0.99816 0.9995
2
                         [,15]
Standard deviation     0.03014
Proportion of Variance 0.00048
Cumulative Proportion  1.00000
```

```
VarCorr(zcp_m6)

 Groups     Name        Std.Dev. Corr
 Player     (Intercept) 0.600525
            c1          0.651932  0.235
            c2          0.381990  0.049  0.149
            c3          0.702563 -0.145  0.189  0.461
            c4          0.455038 -0.468  0.420  0.484  0.698
 Player.1   phase       0.182243
 Player.2   tc1         0.106518
 Player.3   tc2         0.054353
 Player.4   wcdt1       0.073058
 Player.5   wcdt2       0.032152
 Player.6   tod2_1      0.355321
 Player.7   tod2_3      0.195089
 Player.8   dalg        0.190874
 Player.9   tod2_1:dalg 0.314117
 Player.10  tod2_3:dalg 0.425072
 Residual               1.066879

anova(zcp_m6, zcp_m5, zcp_m2)

Data: dat2
Models:
zcp_m6: keb ~ 1 + (tod2_1 + tod2_3) * (phase + tc1 + tc2 + wcdt1 + wcdt2 + c1
+ c2 + c3 + c4 + dalg) + tc1:(c1 + c2 + c3 + c4) + (1 + c1 + c2 + c3 + c4 | P
layer) + ((0 + phase | Player) + (0 + tc1 | Player) + (0 + tc2 | Player) + (0
+ wcdt1 | Player) + (0 + wcdt2 | Player) + (0 + tod2_1 | Player) + (0 + tod2_
3 | Player) + (0 + dalg | Player) + (0 + tod2_1:dalg | Player) + (0 + tod2_3:
dalg | Player))
zcp_m5: keb ~ 1 + (tod2_1 + tod2_3) * (phase + tc1 + tc2 + wcdt1 + wcdt2 + c1
+ c2 + c3 + c4 + dalg) + tc1:(c1 + c2 + c3 + c4) + (1 + c1 + c2 + c3 + c4 | P
layer) + ((0 + phase | Player) + (0 + tc1 | Player) + (0 + tc2 | Player) + (0
+ wcdt1 | Player) + (0 + wcdt2 | Player) + (0 + tod2_1 | Player) + (0 + tod2_
3 | Player) + (0 + game | Player) + (0 + dalg | Player) + (0 + tod2_1:game |
Player) + (0 + tod2_3:game | Player) + (0 + tod2_1:dalg | Player) + (0 + tod2
_3:dalg | Player))
zcp_m2: keb ~ 1 + (tod2_1 + tod2_3) * (phase + tc1 + tc2 + wcdt1 + wcdt2 + c1
+ c2 + c3 + c4 + game + dalg) + (c1 + c2 + c3 + c4) * tc1 + (1 + c1 + c2 + c3
+ c4 | Player) + ((0 + phase | Player) + (0 + tc1 | Player) + (0 + tc2 | Play
er) + (0 + wcdt1 | Player) + (0 + wcdt2 | Player) + (0 + tod2_1 | Player) + (
0 + tod2_3 | Player) + (0 + game | Player) + (0 + dalg | Player) + (0 + tod2_
1:game | Player) + (0 + tod2_3:game | Player) + (0 + tod2_1:dalg | Player) +
(0 + tod2_3:dalg | Player))
       npar   AIC   BIC logLik -2*log(L)  Chisq Df Pr(>Chisq)
zcp_m6   63 24123 24562 -11998     23997
zcp_m5   66 24052 24512 -11960     23920 76.842  3     <2e-16 ***
zcp_m2   69 24047 24528 -11954     23909 11.139  3      0.011 *
---
Signif. codes:  0 '***' 0.001 '**' 0.01 '*' 0.05 '.' 0.1 ' ' 1
```

Answer: Yes. Primarily because of individual differences between players. There is no loss of goodness of fit when we take out only the game-related fixed effects, but keep the game-related VCs.

## Post-hoc LMM: zcp_m2x

To check quadratic trends nested within levels of time-of-day, we include the tc1:(c1+c2+c3+c4) interaction terms in the nested structure. This estimates 8 additional parameters that do not not change the goodness of fit, but allow the check of linear and quadratic trends levels of time-of-day factor.

```
zcp_m2x <-
lmer(keb ~ 1 + (tod2_1 + tod2_3)*(phase+ tc1+tc2 + wcdt1+wcdt2 +
           c1+c2+c3+c4 + game+dalg + tc1:(c1+c2+c3+c4) ) +
           (1 + c1+c2+c3+c4 | Player) +
           (0 + phase +  tc1 + tc2 + wcdt1 + wcdt2 + tod2_1 + tod2_3 + game +
dalg +
            tod2_1:game + tod2_3:game + tod2_1:dalg +tod2_3:dalg || Player),
      dat=dat2, REML=FALSE, control=lmerControl(calc.derivs = FALSE))

ph_zcp_m2x <-
lmer(keb ~ 1 + ToD/(phase + tc1+tc2 + wcdt1+wcdt2 +
           c1+c2+c3+c4 + game+dalg + tc1:(c1+c2+c3+c4) ) +
           (1 + c1+c2+c3+c4 | Player) +
           (0 + phase +  tc1 + tc2 + wcdt1 + wcdt2 + tod2_1 + tod2_3 + game +
dalg +
           tod2_1:game + tod2_3:game + tod2_1:dalg +tod2_3:dalg || Player),
     dat=dat2, REML=FALSE, control=lmerControl(calc.derivs = FALSE))
summary(rePCA(ph_zcp_m2x)) # supported!

$Player
Importance of components:
                         [,1]   [,2]   [,3]    [,4]    [,5]    [,6]    [,7]
Standard deviation     0.8112 0.6318 0.5092 0.40813 0.40055 0.35226 0.31178
Proportion of Variance 0.2945 0.1787 0.1160 0.07455 0.07181 0.05554 0.04351
Cumulative Proportion  0.2945 0.4732 0.5893 0.66382 0.73563 0.79116 0.83467
                          [,8]    [,9]   [,10]   [,11]   [,12]   [,13]   [,14]
Standard deviation     0.30210 0.3016 0.21460 0.21118 0.19961 0.14449 0.13840
Proportion of Variance 0.04085 0.0407 0.02061 0.01996 0.01783 0.00934 0.00857
Cumulative Proportion  0.87552 0.9162 0.93683 0.95679 0.97463 0.98397 0.99254
                         [,15]   [,16]   [,17]   [,18]
Standard deviation     0.09539 0.06628 0.04974 0.02628
Proportion of Variance 0.00407 0.00197 0.00111 0.00031
Cumulative Proportion  0.99662 0.99858 0.99969 1.00000

VarCorr(ph_zcp_m2x)

 Groups     Name        Std.Dev. Corr
 Player     (Intercept) 0.568355
            c1          0.654315  0.193
            c2          0.383183  0.108  0.146
```

```
           c3          0.703312 -0.113  0.185  0.461
           c4          0.456365 -0.451  0.420  0.483  0.696
 Player.1  phase       0.146195
 Player.2  tc1         0.100763
 Player.3  tc2         0.052544
 Player.4  wcdt1       0.070019
 Player.5  wcdt2       0.027765
 Player.6  tod2_1      0.319120
 Player.7  tod2_3      0.223080
 Player.8  game        0.210858
 Player.9  dalg        0.226692
 Player.10 tod2_1:game 0.372103
 Player.11 tod2_3:game 0.423114
 Player.12 tod2_1:dalg 0.318555
 Player.13 tod2_3:dalg 0.431130
 Residual              1.056344
```

```
anova(zcp_m2x, ph_zcp_m2x)
```

```
Data: dat2
Models:
zcp_m2x: keb ~ 1 + (tod2_1 + tod2_3) * (phase + tc1 + tc2 + wcdt1 + wcdt2 + c
1 + c2 + c3 + c4 + game + dalg + tc1:(c1 + c2 + c3 + c4)) + (1 + c1 + c2 + c3
+ c4 | Player) + ((0 + phase | Player) + (0 + tc1 | Player) + (0 + tc2 | Play
er) + (0 + wcdt1 | Player) + (0 + wcdt2 | Player) + (0 + tod2_1 | Player) + (
0 + tod2_3 | Player) + (0 + game | Player) + (0 + dalg | Player) + (0 + tod2_
1:game | Player) + (0 + tod2_3:game | Player) + (0 + tod2_1:dalg | Player) +
(0 + tod2_3:dalg | Player))
ph_zcp_m2x: keb ~ 1 + ToD/(phase + tc1 + tc2 + wcdt1 + wcdt2 + c1 + c2 + c3 +
c4 + game + dalg + tc1:(c1 + c2 + c3 + c4)) + (1 + c1 + c2 + c3 + c4 | Player
) + ((0 + phase | Player) + (0 + tc1 | Player) + (0 + tc2 | Player) + (0 + wc
dt1 | Player) + (0 + wcdt2 | Player) + (0 + tod2_1 | Player) + (0 + tod2_3 |
Player) + (0 + game | Player) + (0 + dalg | Player) + (0 + tod2_1:game | Play
er) + (0 + tod2_3:game | Player) + (0 + tod2_1:dalg | Player) + (0 + tod2_3:d
alg | Player))
           npar   AIC   BIC logLik -2*log(L) Chisq Df Pr(>Chisq)
zcp_m2x      77 24053 24590 -11949     23899
ph_zcp_m2x   77 24053 24590 -11949     23899 1e-04  0
```

```
compare_parameters(zcp_m2, ph_zcp_m2x)
```

```
Parameter                 |                zcp_m2 |             ph_zcp_m2x
--------------------------------------------------------------------------
(Intercept)               |     3.76 ( 3.50,  4.02) |      3.76 ( 3.50,  4.02)
dalg                      |    -0.04 (-0.20,  0.13) |
tod2 1                    |     0.74 ( 0.50,  0.98) |
tod2 3                    |     0.37 ( 0.15,  0.59) |
phase                     |     0.14 ( 0.04,  0.24) |
tc1                       |     0.05 ( 0.00,  0.10) |
tc2                       |     0.05 ( 0.02,  0.08) |
wcdt1                     |    -0.04 (-0.07,  0.00) |
wcdt2                     |     0.01 (-0.01,  0.03) |
```

| | | |
|---|---|---|
| c1 | 0.90 ( 0.58, 1.22) | |
| c2 | 0.95 ( 0.77, 1.14) | |
| c3 | 0.78 ( 0.46, 1.10) | |
| c4 | 8.03e-03 (-0.21, 0.23) | |
| game | -0.06 (-0.20, 0.09) | |
| tod2 3 × tc1 | 0.07 ( 0.02, 0.13) | |
| tod2 1 × phase | 0.27 ( 0.07, 0.47) | |
| tod2 1 × tc1 | 0.01 (-0.06, 0.08) | |
| tod2 1 × tc2 | -0.02 (-0.06, 0.01) | |
| tod2 1 × wcdt1 | 0.05 (-0.02, 0.11) | |
| tod2 1 × wcdt2 | -0.06 (-0.09, -0.03) | |
| tod2 1 × c1 | 0.68 ( 0.31, 1.04) | |
| tod2 1 × c2 | -0.38 (-0.60, -0.15) | |
| tod2 1 × c3 | -0.37 (-0.58, -0.16) | |
| tod2 1 × c4 | 0.42 ( 0.16, 0.68) | |
| tod2 1 × game | -0.16 (-0.48, 0.17) | |
| tod2 1 × dalg | -0.63 (-0.99, -0.28) | |
| tod2 3 × phase | 0.48 ( 0.28, 0.68) | |
| tc1 × c4 | -0.05 (-0.12, 0.03) | |
| tod2 3 × tc2 | 0.06 ( 0.03, 0.09) | |
| tod2 3 × wcdt1 | -0.02 (-0.06, 0.03) | |
| tod2 3 × wcdt2 | -0.04 (-0.07, -0.01) | |
| tod2 3 × c1 | 0.09 (-0.25, 0.43) | |
| tod2 3 × c2 | -0.65 (-0.86, -0.44) | |
| tod2 3 × c3 | 0.36 ( 0.16, 0.55) | |
| tod2 3 × c4 | -0.45 (-0.69, -0.21) | |
| tod2 3 × game | -0.59 (-0.93, -0.24) | |
| tod2 3 × dalg | -0.86 (-1.22, -0.49) | |
| tc1 × c1 | -0.08 (-0.19, 0.03) | |
| tc1 × c2 | -0.10 (-0.17, -0.04) | |
| tc1 × c3 | -0.21 (-0.27, -0.15) | |
| ToD [Morning] × wcdt1 | | -0.03 (-0.07, 0.02) |
| ToD2-1 | | 0.74 ( 0.50, 0.98) |
| ToD3-2 | | -0.37 (-0.59, -0.15) |
| ToD [Night] × phase | | 0.12 (-0.03, 0.27) |
| ToD [Morning] × phase | | 0.39 ( 0.23, 0.55) |
| ToD [Day] × phase | | -0.09 (-0.24, 0.06) |
| ToD [Night] × tc1 | | 0.07 ( 0.00, 0.13) |
| ToD [Morning] × tc1 | | 0.08 ( 0.02, 0.15) |
| ToD [Day] × tc1 | | 7.72e-03 (-0.05, 0.06) |
| ToD [Night] × tc2 | | 0.09 ( 0.05, 0.12) |
| ToD [Morning] × tc2 | | 0.06 ( 0.03, 0.10) |
| ToD [Day] × tc2 | | 2.64e-03 (-0.03, 0.03) |
| ToD [Night] × wcdt1 | | -0.07 (-0.13, -0.01) |
| ToD [Day] × c3 | | 0.40 ( 0.07, 0.74) |
| ToD [Day] × wcdt1 | | -8.91e-03 (-0.05, 0.04) |
| ToD [Night] × wcdt2 | | 0.04 ( 0.01, 0.06) |
| ToD [Morning] × wcdt2 | | -0.02 (-0.05, 0.00) |
| ToD [Day] × wcdt2 | | 0.02 ( 0.00, 0.05) |
| ToD [Night] × c1 | | 0.48 ( 0.09, 0.87) |
| ToD [Morning] × c1 | | 1.20 ( 0.81, 1.59) |

```
ToD [Day] × c1              |                          |       1.13 ( 0.77,  1.50)
ToD [Night] × c2            |                          |       1.00 ( 0.76,  1.23)
ToD [Morning] × c2          |                          |       0.59 ( 0.36,  0.82)
ToD [Day] × c2              |                          |       1.25 ( 1.03,  1.46)
ToD [Night] × c3            |                          |       1.16 ( 0.81,  1.50)
ToD [Morning] × c3          |                          |       0.76 ( 0.41,  1.10)
ToD [Night] × tc1 × c2      |                          |      -0.14 (-0.28, -0.01)
ToD [Night] × c4            |                          |      -0.41 (-0.68, -0.14)
ToD [Morning] × c4          |                          |   8.18e-03 (-0.27,  0.28)
ToD [Day] × c4              |                          |       0.47 ( 0.22,  0.73)
ToD [Night] × game          |                          |      -0.15 (-0.39,  0.10)
ToD [Morning] × game        |                          |      -0.30 (-0.55, -0.06)
ToD [Day] × game            |                          |       0.28 ( 0.02,  0.54)
ToD [Night] × dalg          |                          |       0.10 (-0.18,  0.38)
ToD [Morning] × dalg        |                          |      -0.54 (-0.79, -0.28)
ToD [Day] × dalg            |                          |       0.32 ( 0.05,  0.60)
ToD [Night] × tc1 × c1      |                          |      -0.11 (-0.33,  0.11)
ToD [Morning] × tc1 × c1    |                          |      -0.26 (-0.48, -0.04)
ToD [Day] × tc1 × c1        |                          |       0.02 (-0.13,  0.17)
ToD [Morning] × tc1 × c3    |                          |      -0.12 (-0.25,  0.00)
ToD [Morning] × tc1 × c2    |                          |      -0.01 (-0.15,  0.12)
ToD [Day] × tc1 × c2        |                          |      -0.12 (-0.22, -0.03)
ToD [Night] × tc1 × c3      |                          |      -0.24 (-0.37, -0.12)
ToD [Day] × tc1 × c4        |                          |  -6.26e-03 (-0.11,  0.10)
ToD [Day] × tc1 × c3        |                          |      -0.23 (-0.32, -0.14)
ToD [Night] × tc1 × c4      |                          |      -0.09 (-0.25,  0.06)
ToD [Morning] × tc1 × c4    |                          |      -0.09 (-0.24,  0.07)
-----------------------------------------------------------------------------
Observations                |                     7908 |                   7908
```

# Appendix

```
sessionInfo()
```

```
R version 4.5.0 (2025-04-11)
Platform: aarch64-apple-darwin20
Running under: macOS Sequoia 15.5

Matrix products: default
BLAS:   /Library/Frameworks/R.framework/Versions/4.5-arm64/Resources/lib/libR
blas.0.dylib
LAPACK: /Library/Frameworks/R.framework/Versions/4.5-arm64/Resources/lib/libR
lapack.dylib;  LAPACK version 3.12.1

locale:
[1] en_US.UTF-8/en_US.UTF-8/en_US.UTF-8/C/en_US.UTF-8/en_US.UTF-8

time zone: Europe/Berlin
tzcode source: internal
```

```
attached base packages:
[1] stats     graphics  grDevices utils     datasets  methods   base

other attached packages:
 [1] cowplot_1.1.3      haven_2.5.4        lubridate_1.9.4    forcats_1.0.0
 [5] stringr_1.5.1      dplyr_1.1.4        purrr_1.0.4        readr_2.1.5
 [9] tidyr_1.3.1        tibble_3.2.1       ggplot2_3.5.2      tidyverse_2.0.0
[13] remef_1.0.7        lme4_1.1-37        Matrix_1.7-3       see_0.11.0
[17] report_0.6.1       parameters_0.25.0  performance_0.13.0 modelbased_0.10
.0
[21] insight_1.2.0      effectsize_1.0.0   datawizard_1.0.2   correlation_0.8
.7
[25] bayestestR_0.15.3  easystats_0.7.4

loaded via a namespace (and not attached):
 [1] gtable_0.3.6       xfun_0.52          lattice_0.22-7     tzdb_0.5.0
 [5] vctrs_0.6.5        tools_4.5.0        Rdpack_2.6.4       generics_0.1.3
 [9] pkgconfig_2.0.3    RColorBrewer_1.1-3 lifecycle_1.0.4    compiler_4.5.0
[13] farver_2.1.2       htmltools_0.5.8.1  yaml_2.3.10        pillar_1.10.2
[17] nloptr_2.2.1       MASS_7.3-65        reformulas_0.4.1   boot_1.3-31
[21] nlme_3.1-168       tidyselect_1.2.1   digest_0.6.37      mvtnorm_1.3-3
[25] stringi_1.8.7      splines_4.5.0      fastmap_1.2.0      grid_4.5.0
[29] cli_3.6.5          magrittr_2.0.3     withr_3.0.2        scales_1.4.0
[33] estimability_1.5.1 timechange_0.3.0   rmarkdown_2.29     emmeans_1.10.7
[37] hms_1.1.3          coda_0.19-4.1      evaluate_1.0.3     knitr_1.50
[41] rbibutils_2.3      rlang_1.1.6        Rcpp_1.0.14        xtable_1.8-4
[45] glue_1.8.0         rstudioapi_0.17.1  minqa_1.2.8        jsonlite_2.0.0
[49] R6_2.6.1
```
